# Biological Effects on μ-Receptors Affinity and Selectivity of Arylpropenyl Chain Structural Modification on Diazatricyclodecane Derivatives

**DOI:** 10.3390/molecules26185448

**Published:** 2021-09-07

**Authors:** Sandra Piras, Gabriele Murineddu, Giovanni Loriga, Antonio Carta, Enrica Battistello, Stefania Merighi, Stefania Gessi, Paola Corona, Battistina Asproni, Roberta Ibba, Veronika Temml, Daniela Schuster, Gérard Aimè Pinna

**Affiliations:** 1 Department of Chemistry and Pharmacy, University of Sassari, via F. Muroni 23/A, 07100 Sassari, Italy; muri@uniss.it (G.M.); acarta@uniss.it (A.C.); pcorona@uniss.it (P.C.); asproni@uniss.it (B.A.); ribba@uniss.it (R.I.); pinger@uniss.it (G.A.P.); 2 Institute of Biomolecular Chemistry, National Research Council, Traversa La Crucca 3, 07100 Sassari, Italy; giovanni.loriga@cnr.it; 3 Department of Morphology, Surgery and Experimental Medicine, University of Ferrara, 44121 Ferrara, Italy; bttnrc@unife.it (E.B.); stefania.merighi@unife.it (S.M.); stefania.gessi@unife.it (S.G.); 4 Department of Pharmaceutical Chemistry, Paracelsus Medical University Salzburg, Strubergasse 21, 5020 Salzburg, Austria; veronika.temml@uibk.ac.at (V.T.); daniela.schuster@pmu.ac.at (D.S.)

**Keywords:** μ-receptors affinity, analgesic activity, 9,10-diazatricyclo[4.2.1.1^2,5^]decane (DTD), 2,7-diazatricyclo[4.4.0.0^3,8^]decane, DTD derivatives, rigid benzo-condensed structure

## Abstract

Opioid analgesics are clinically used to relieve severe pain in acute postoperative and cancer pain, and also in the long term in chronic pain. The analgesic action is mediated by μ-, δ-, and κ-receptors, but currently, with few exceptions for k-agonists, μ-agonists are the only ones used in therapy. Previously synthesized compounds with diazotricyclodecane cores (DTDs) have shown their effectiveness in binding opioid receptors. Fourteen novel diazatricyclodecanes belonging to the 9-propionyl-10-substituted-9,10-diazatricyclo[4.2.1.1^2,5^]decane (compounds **20**–**23**, **53**, **57** and **59**) and 2-propionyl-7-substituted-2,7-diazatricyclo[4.4.0.0^3,8^]decane (compounds **24**–**27**, **54**, **58** and **60**) series, respectively, have been synthesized and their ability to bind to the opioid μ-, δ- and κ-receptors was evaluated. Five of these derivatives, compounds **20**, **21**, **24**, **26** and **53**, showed μ-affinity in the nanomolar range with a negligible affinity towards δ- and κ-receptors and high μ-receptor selectivity. The synthesized compounds showed μ-receptor selectivity higher than those of previously reported methylarylcinnamyl analogs.

## 1. Introduction

Pain is an essential defense the human body activates as a result of noxious stimuli. It can be defined as a sensorial and emotional experience correlated to tissue damage. The perception of pain as such is subjective when considering severity and tolerance. Pain is classified into acute and chronic. Acute pain is mediated by nociceptors activation in the tissue damage site, it is transient, and intensity decreases with the healing of the injury that caused it. Chronic pain (CNP), on the other hand, lasts longer than six months and may continue after the injury or illness has been treated. Therefore, CNP is considered a pathologic process involving the somato-sensory system, caused by abnormal processing of stimuli arriving from cellular damage location in the central nervous system (CNS) or peripheral nervous system (PNS) [1]. Chronic neuropathic pain (CNP) can result from surgical treatment, diabetes, spinal injury, multiple sclerosis and several other conditions. It affects a high percentage of adults globally and the treatment of chronic pain is a crucial issue worldwide. Opioid analgesics are the oldest and still the most potent drugs widely used for the treatment of acute and chronic pain. Their antinociceptive activity is exerted by interacting with central and peripheral opioid receptors [2], a large superfamily of G protein-coupled receptors (GPCRs). Agonist-receptor interaction leads to the adenylate cyclase inhibition that results in cytoplasmic cAMP decrease. It also brings to an opening of the potassium channels and an inhibition of voltage-gated calcium channels opening. The membrane permeability impairment reduces neuronal excitability and transmitters release leading to an overall analgesic effect and a raised pain threshold [2]. The CNS opioid receptors can be classified into mu (μOR), delta (δOR), and kappa (κOR) receptors, and they show a high rate of structure homology among isoforms. Endogenous opioid peptides, endorphins, dynorphins and enkephalins target the opioid receptors. Recently the μOR pathway has been discovered to be modulated by a nociceptin/orphanin FQ (N/OFQ) peptide and its related nociceptin opioid peptide (NOP) receptor [3]. The μORs are mainly expressed in the cerebellum, caudate nucleus, nucleus accumbens, amygdala, cerebral cortex and spinal cord [4,5]. The expression of μOR within the descending pain modulatory pathway, which includes the medulla locus coeruleus, the dorsal horn of the spinal cord and the periaqueductal gray, contribute to opioid-induced antinociception as well as to opioid resistance [6,7,8,9]. The δORs are also expressed in spinal cord as well as in basal ganglia and mesolimbic system [4,5,10,11]. While κOR are largely expressed throughout the CNS, inter alia the mesolimbic system, spinal cord, hypothalamus, amygdala. κORs are mainly expressed in the presynaptic dopaminergic membranes and κOR agonists inhibit presynaptic neurotransmitter release. On the other hand, δOR agonists were proved to deliver antinociceptive, anxiolytic and antidepressant-like effects in animal models [4,5,12].

**Figure 1:**
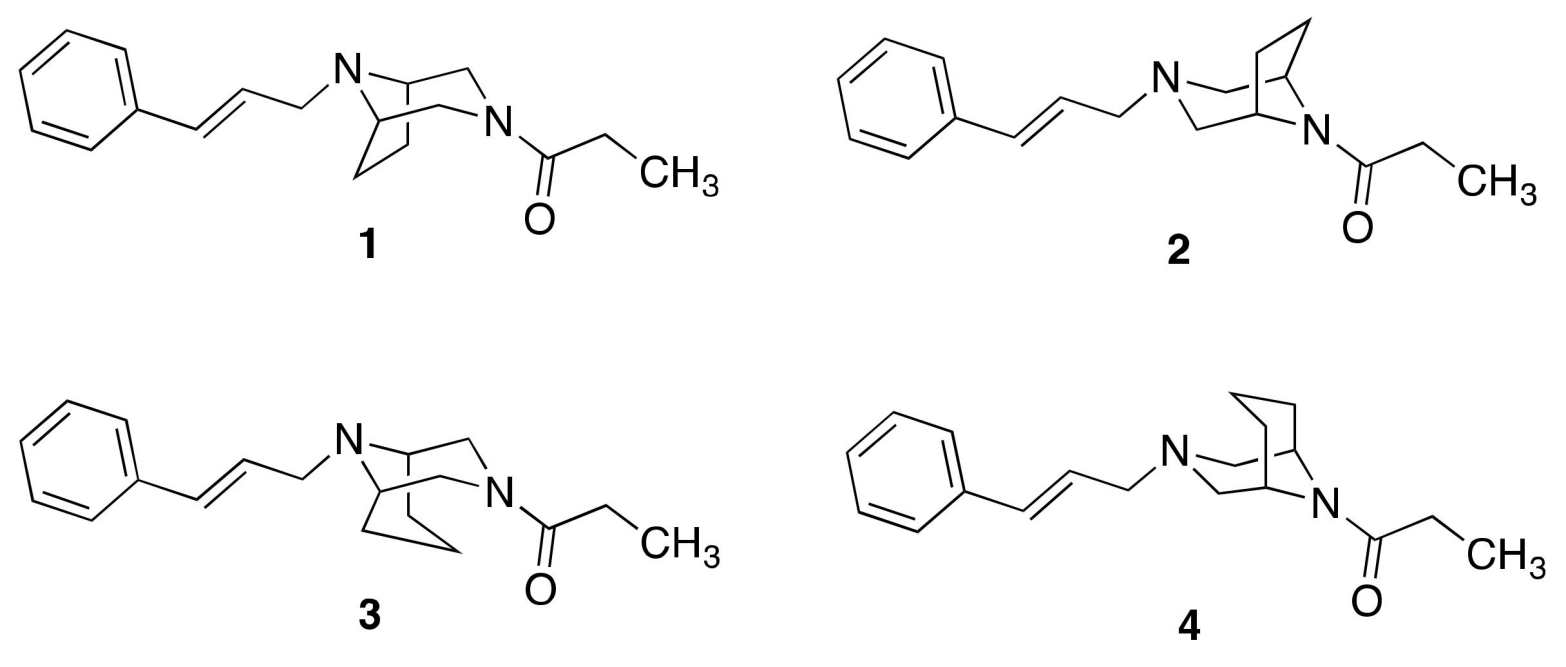
3,8-Diazabicyclo[3.2.1]octanes (**1**,**2**) and 3,9-diazabicyclo[3.2.1]nonanes (**3**,**4**).

The analgesic effect of opioid derivatives is often associated with severe side effects [13,14]. The side effects of μOR agonists comprise mental clouding, sedation, respiratory depression, but also euphoria, antidiuresis, nausea, bradycardia and histamine release [15], while selective κOR agonists produce antinociceptive effects and sedation, accompanied by adverse anxiogenic and hallucinogenic effects that have limited the clinical use of these agonists [16]. Despite the fact no δOR agonists are actually used in therapy, the selective activation of δ opioid receptors has been studied and proved to be associated with antinociceptive effects along with anxiolytic-like and antidepressant-like effects. The resulting emotional response is convenient because of the recurrent association of mood turmoil with chronic pain [15,17]. A recently developed rational approach to opioid therapy aims to combine different opioid receptors agonists to reach the therapeutic effect while negating the side effects [16,18]. The urgency to develop new drugs to treat chronic pain while minimizing the side effects, therefore, comes through the design and synthesis of novel opioid receptor ligands. In the 1960s a research program aimed at identifying new analgesic structures led to obtaining some 3,8-diazabicyclo[3.2.1]octane derivatives (DBOs) with interesting central analgesic activity [19,20,21]. These derivatives (compounds **1**,**2**, Figure 1) possessed powerful and selective μ-affinity and a significant analgesic activity. 

Structure-activity relationship (SAR) studies on these templates have emphasized that the cinnamyl portion played a pivotal role in μ-affinity. To further evaluate the influence of the endoethanic bridge of 3,8-diazatricyclo[3.2.1]octane (DBO) structure on the receptor-drug complex stability, it was considered useful to evaluate its homologation to an endopropanic bridge to give the 3,9-diazabicyclo[3.2.1]nonane (DBN) scaffold (compounds **3** and **4**, Figure 1). The in vitro data of diazabicycloctane and diazabicyclononane analogs showed that the dimensions of the loop induced different effects on the corresponding ligands [22,23]. Nevertheless, the endoethanic and endopropanic bridges played a pivotal role in the interaction with the μ-receptor.

**Figure 2:**
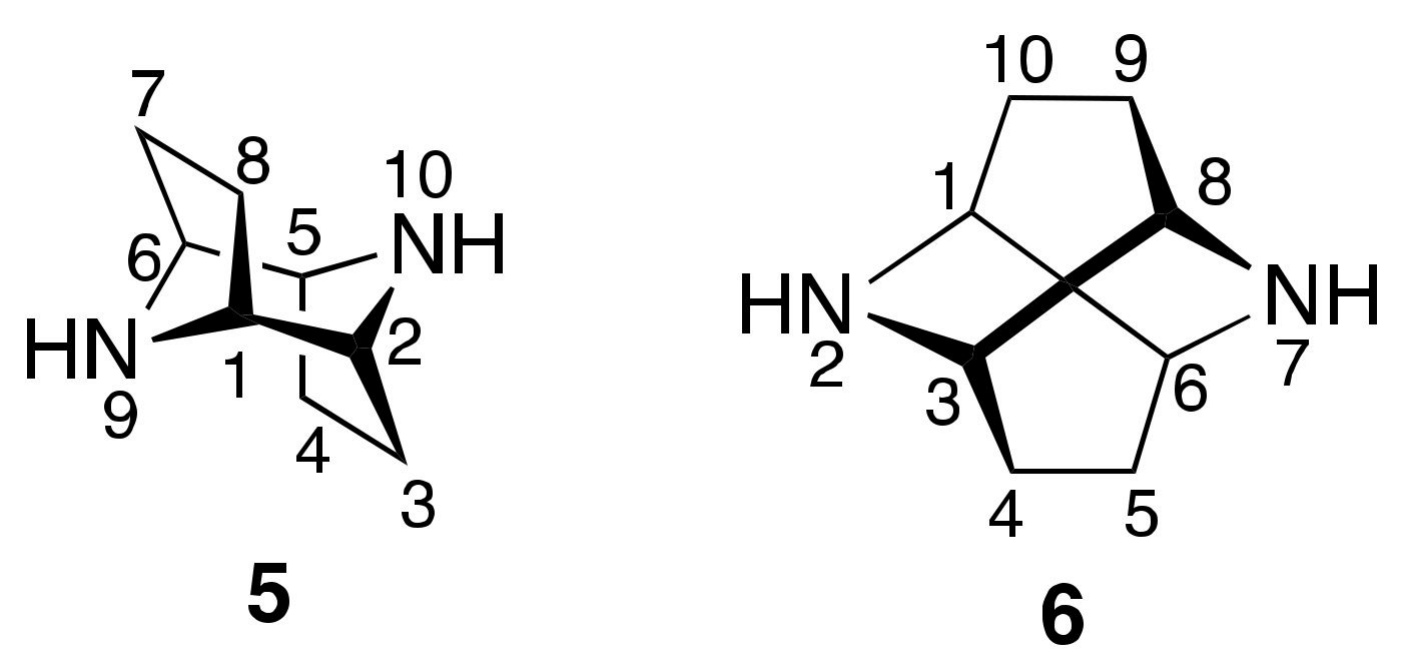
9,10-Diazatricyclo[4.2.1.1^2,5^]decane (**5**) and 2,7-diazatricyclo[4.4.0.0^3,8^]decane (**6**).

Therefore, assuming that the introduction of a second endoethanic bridge on the piperazine portion of the DBO could be a powerful feature for the interaction with the μ-receptor, two novel cores were synthesized: the 9,10-diazatricyclo[4.2.1.1^2,5^]decane (DTD) moiety **5**, containing two bridges between atoms 1,6 and 2,5 of DTD and its isomer 2,7-diazatricyclo[4.4.0.0^3,8^]decane **6**, bridged on positions 1,8 and 3,6 (Figure 2).

**Figure 3:**
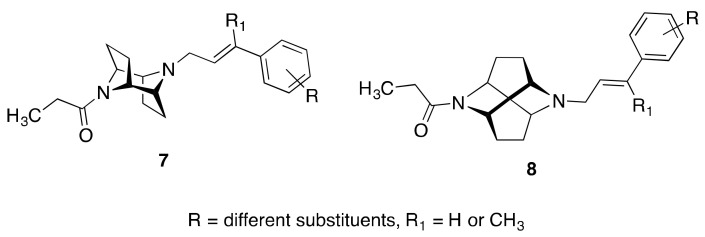
*N*-Functionalization of DTDs.

The appropriate replacement on the nitrogen atoms of the two DTDs, both with the propionyl group and the cinnamyl chain, led to the identification of compounds typified by general structures **7** and **8** [24] (Figure 3), whose in vitro binding studies indicated a significant selectivity towards μ receptors, concerning to κ and δ, for both series of DTDs, some of which, for R_1_ = CH_3_, with μ-affinity values (*K*_i_ = 1.29 − 4.07 nM) comparable to morphine (*K*_i_ = 1.07 nM) [24].

In order to better define the influence of the cinnamyl side chain for the interaction of **7** and **8** with the receptor site, two different modifications were designed: the incorporation of a CH_3_ group on the cinnamyl chain both into a rigid benzocondensed structure and into a bicyclic heteroaromatic system.

**Figure 4:**
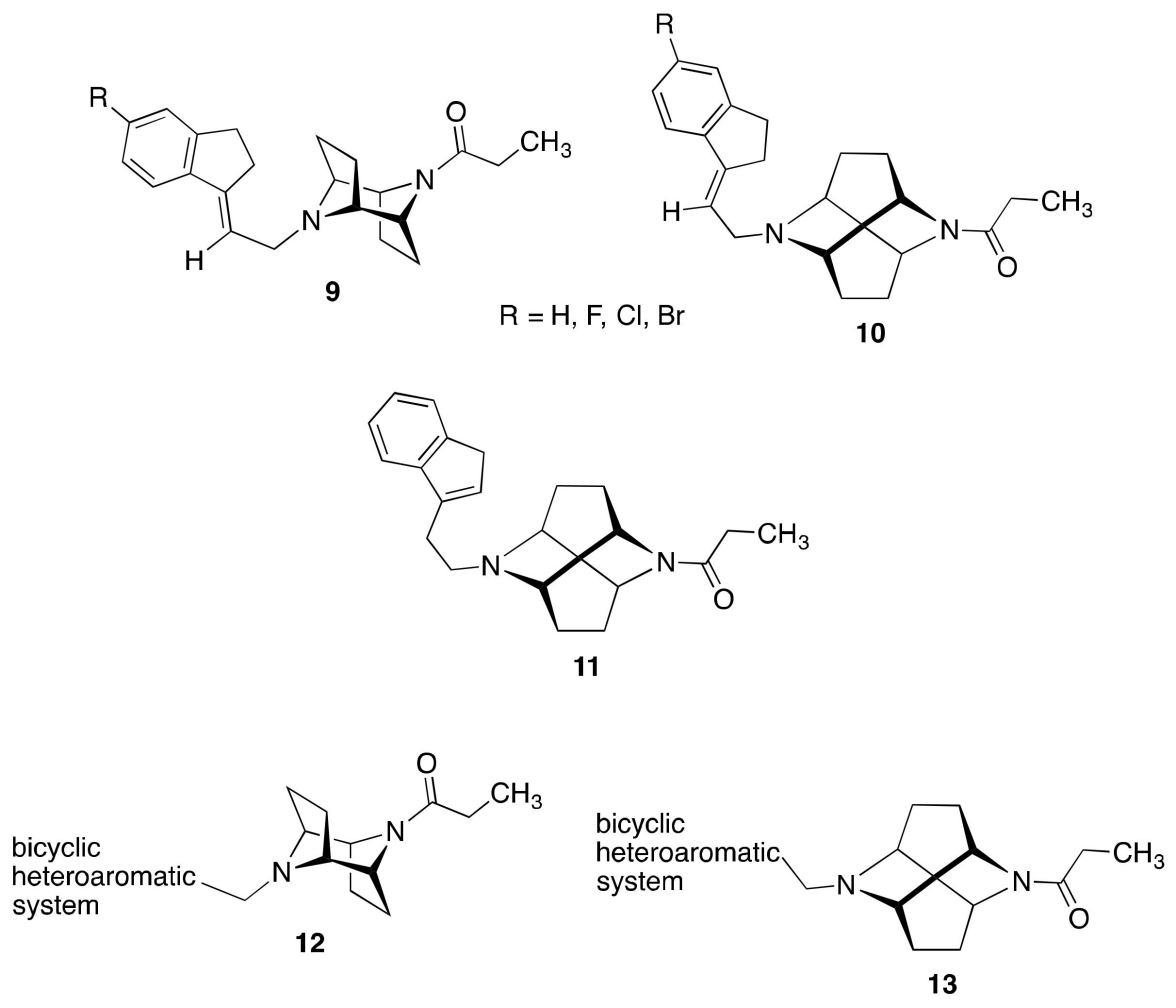
Designed DTD derivatives.

Therefore, we started with the substitution of the methylcinnamyl chain with an indenylidenic group to afford derivatives of general structures **9** and **10** (Figure 4) by reacting the appropriate aldehydes with the DTD. Unexpectedly, and only for the condensation between the aldehydes and the 2-propionyl-2,7-diazatricylo[4.4.0.0^3,8^]decane bicyclic system, the *endo* derivative **11** was also obtained (Figure 4).

As the second step of our project, we planned the introduction of a heteroaromatic system that mimics the cinnamyl chain, synthesizing derivatives of general structures **12** and **13** (Figure 4).

In this paper we report the synthesis and the binding data against μ-, δ- and κ-receptors of novel DTD derivatives, compounds **20**–**27**, **53**–**54** and **57**–**60**, reported in Table 1, in which the side cinnamyl chain is forced into a limited number of conformations.

## 2. Results and Discussion

### 2.1. Chemistry

**Scheme 1:**
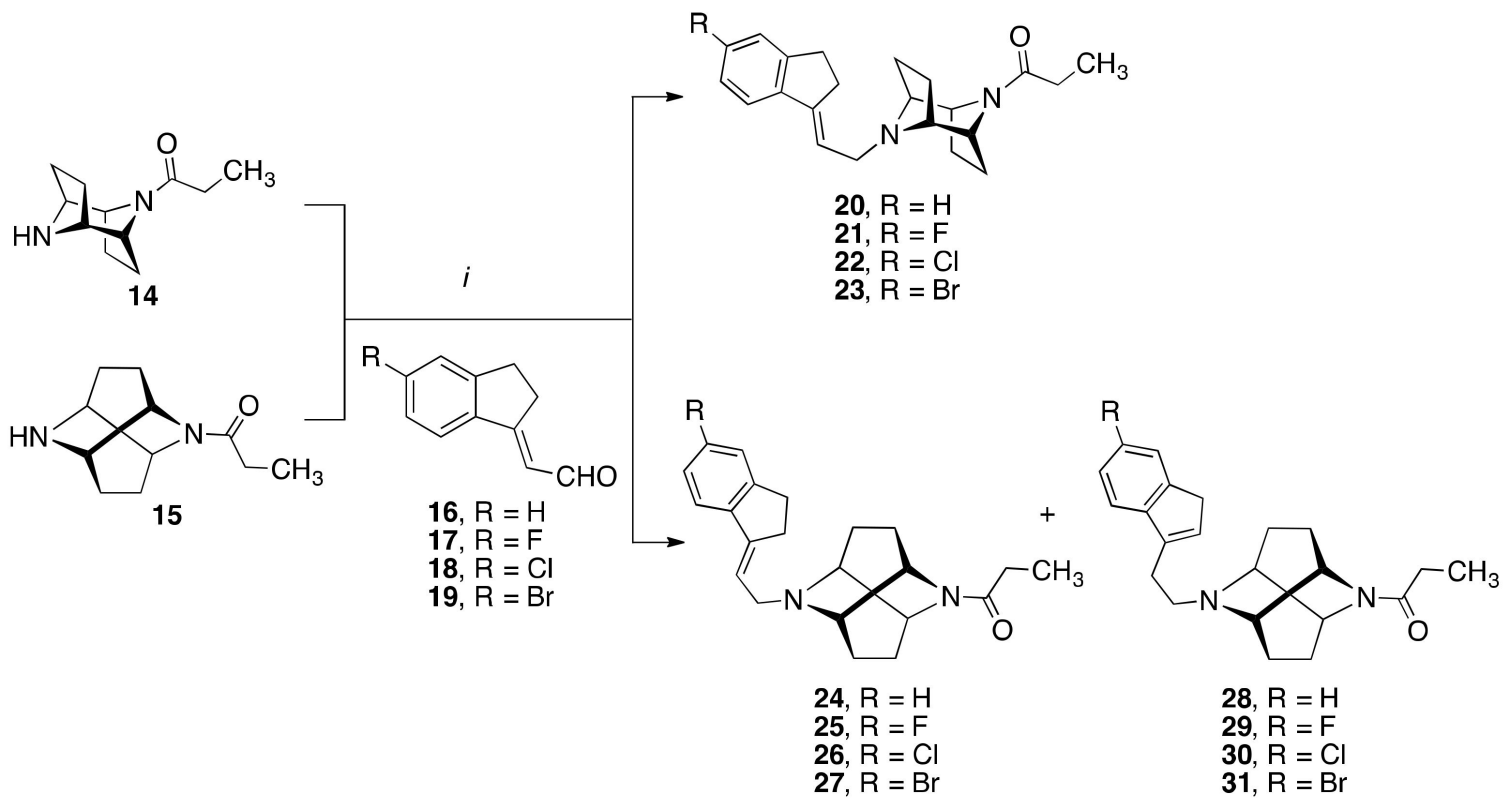
Synthetic route to obtain derivatives **20**–**31**. *Reagents and conditions*: (*i*) NaBH_3_CN, MeOH, AcOH, 7 h, r.t.

**Scheme 2:**
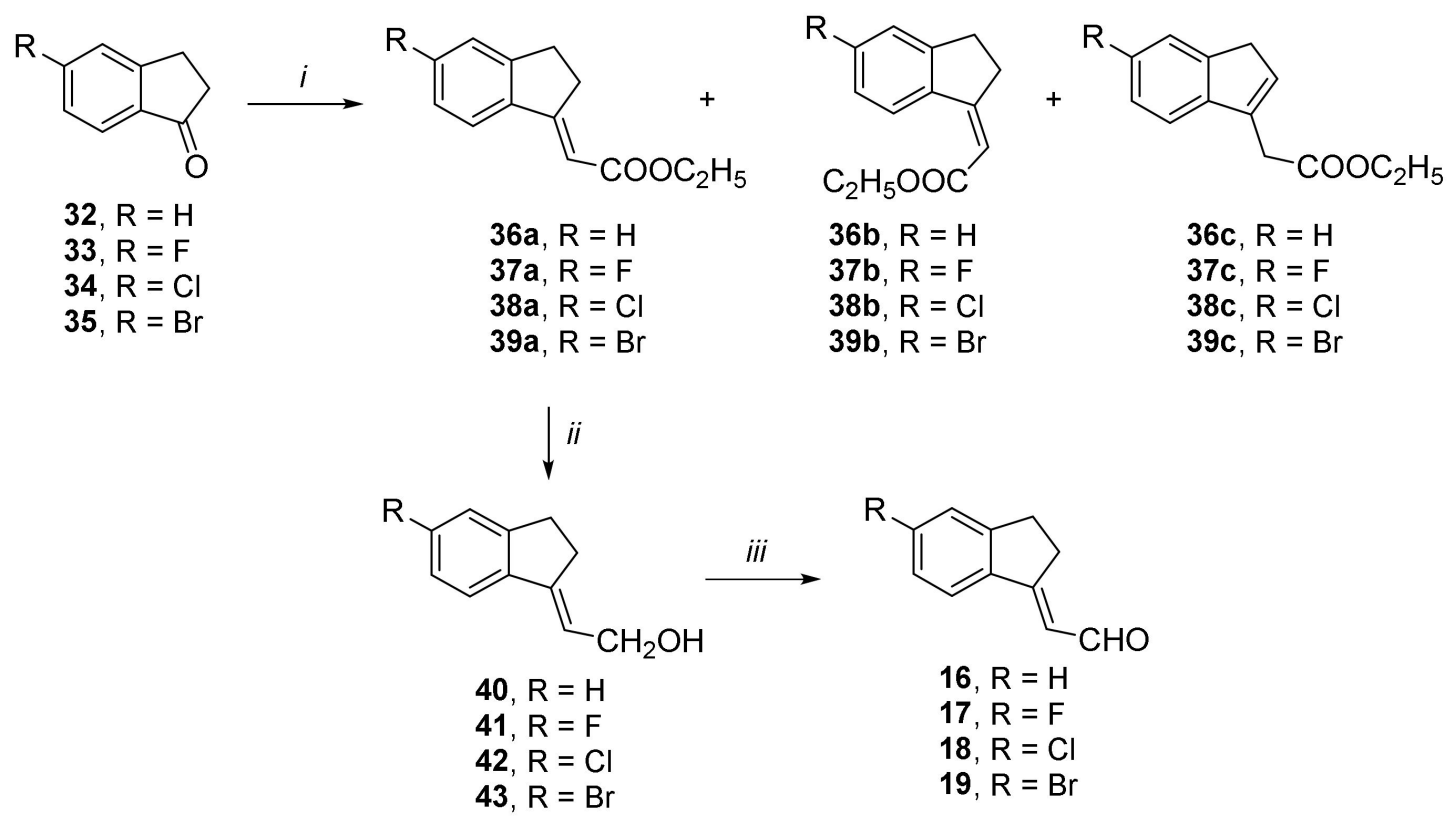
Synthetic route to obtain derivatives **16**–**19**. *Reagents and conditions*: (*i*) NaH, triethylphosponacetate (TEFA), anhydrous toluene and N_2_ at 4 °C; (*ii*) DIBAL-H, anhydrous toluene and N_2_ at 0 °C; (*iii*) MnO_2_, r.t.

**Scheme 3:**
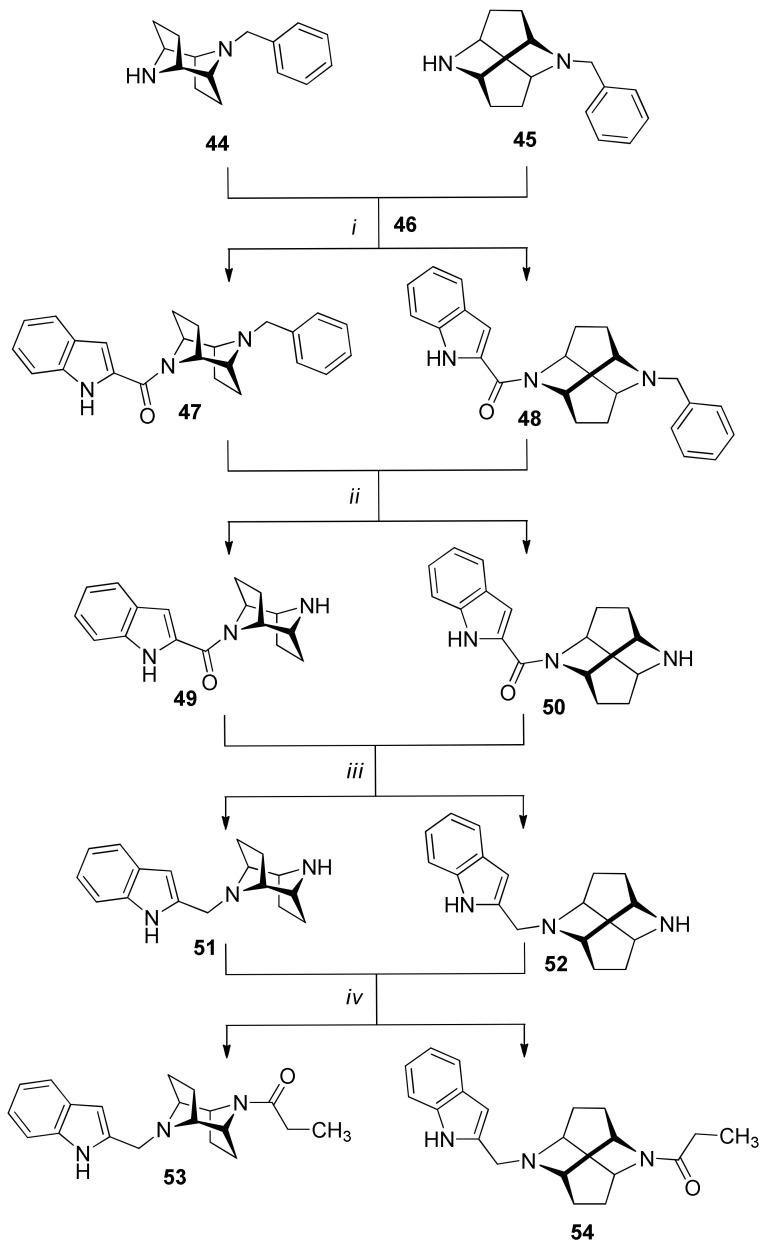
Synthetic route to obtain derivatives **53**,**54**. *Reagents and conditions*: (*i*) 1*H*-indole-2-carbonyl chloride (**46)**, triethylamine (TEA), anhydrous toluene and N_2_ at room temperature; (*ii*) H_2_, 10% Pd/C, ethanol at 60 °C; (*iii*) LiAlH_4_, tetrahydrofuran (THF), 0 °C; (*iv*) propionic anhydride, dichloromethane, 0 °C.

**Scheme 4:**
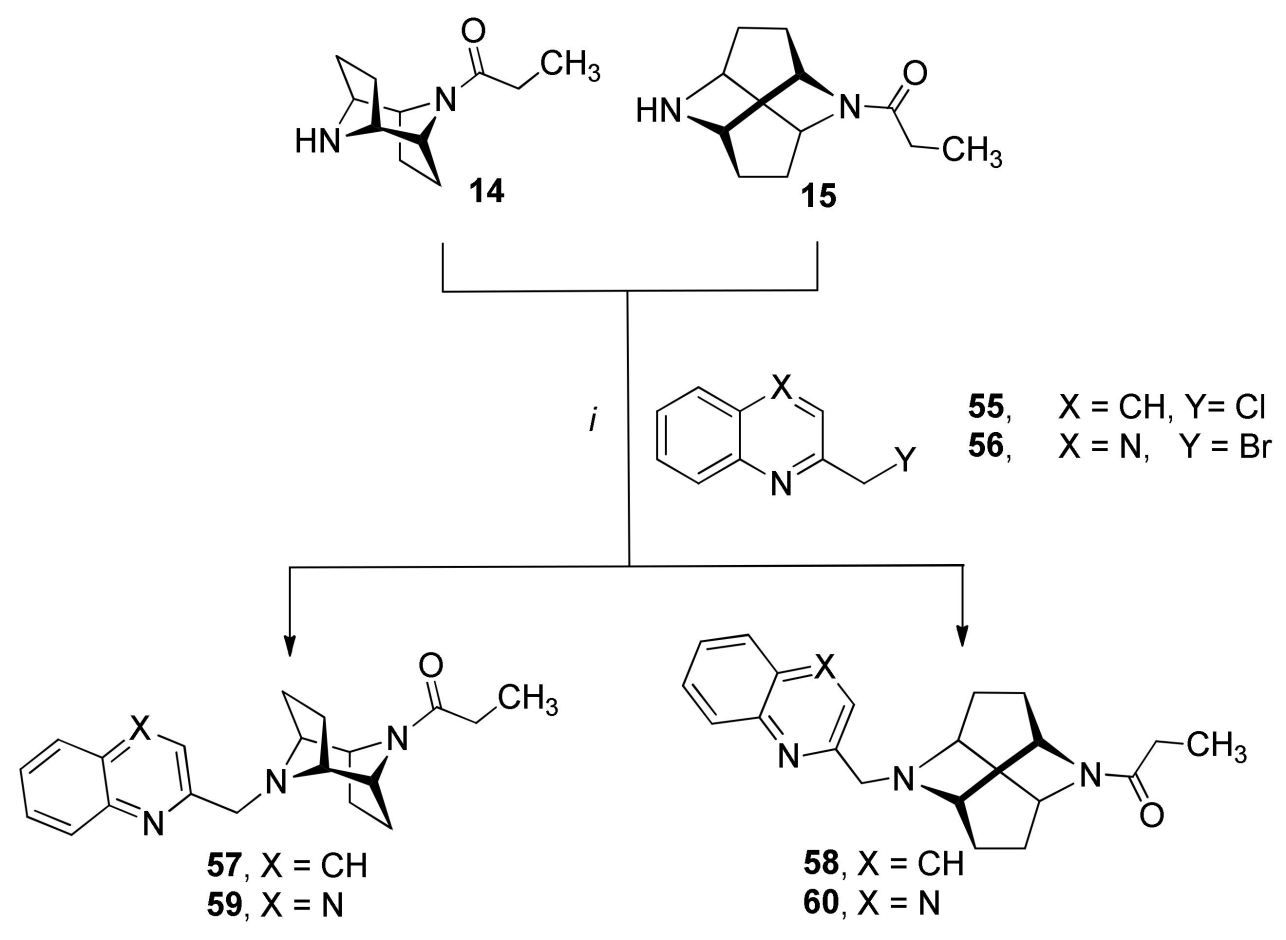
Synthetic route to obtain derivatives **57**–**60**. *Reagents and conditions*: (*i*) Acetone, K_2_CO_3_, 60 °C.

Final derivatives **20**–**27**, were synthesized as reported in Scheme 1, by using aldehydes synthesized as described in Scheme 2. Compounds **53**–**54** and **57**–**60** were synthesized as reported in Scheme 3 and Scheme 4, respectively. The amine intermediates 9-propionyl-9,10-diazatricyclo[4.2.1.1^2,5^]- decane (**14**) and 2-propionyl-2,7-diazatricyclo[4.4.0.0^3,8^]decane (**15**), synthesized following the literature [25], were used as starting compounds. A first attempt to prepare derivatives **20**–**27** provided for a synthetic approach similar to that used for both DBO and DBN series, consisting of a simple alkylation of amines **14** and **15** with the required alkyl chlorides, but unexpectedly this condensation failed. Therefore, we planned a sodium cyanoborohydride reductive amination between a slight molar excess of amine **14** and the appropriate aldehyde **16**–**19** in methanol, in the presence of a catalytic amount of acetic acid. Subsequent purification by flash chromatography of the crude products gave the desired compounds **20**–**23** (Scheme 1).

The same reaction with the amine **15** led to a mixture of compounds, whose separation by flash chromatography, led us to obtain two fractions in a 2:1 ratio: the former, the expected *exo* compounds **24**–**27**, whereas the latter corresponded to the the unexpected *endo* derivatives **28**–**31** (Scheme 1).

The three-step synthetic route for the preparation of aldehydes **16**–**19** is depicted in Scheme 2. The commercially available indanones **32**–**35** underwent a Horner-Wadsworth-Emmons reaction to afford the corresponding esters **36**–**39**, in accordance with literature for **35** [26]. This synthetic pathway, providing the use of triethyl phosphonacetate (TEFA) and sodium hydride in anhydrous toluene, under a N_2_ atmosphere, afforded a mixture of the *exo-E* (**36a**–**39a**), *exo-Z* (**36b**–**39b**) and *endo* (**36c**–**39c**) isomer esters and a 25–30% amount of unreacted starting product. The *exo-E* isomers **36a**–**39a**, separated by flash chromatography, were reduced to the respective alcohols **40**–**43** with diisobutylaluminium hydride (DIBAL-H) in dry toluene under a N_2_ atmosphere at 0 °C. The treatment of **40**–**43** with MnO_2_ at room temperature gave the aldehydes **16**–**19** in good yields.

The synthesis of compounds **53** and **54** (Scheme 3) provides an inversion of *N*-alkylation sequence if compared with the previous one reported for derivatives **20**–**27**. The benzyl derivatives of 9,10-diazatricyclo[4.2.1.1^2,5^]decane (**44**) and 2,7-diazatricyclo[4.4.0.0^3,8^]decane (**45**) [25], were reacted with the indole carbonyl chloride **46**, obtained by thionyl chloride treatment of the corresponding commercial acid, to give derivatives **47** and **48**, respectively. Their *N*-debenzylation with hydrogen on 10% palladium on carbon afforded **49** and **50**, whose reduction with lithium aluminum hydride in tetrahydrofuran gave compounds **51** and **52**. The final acylation with propionic anhydride yielded derivatives **53** and **54**.

*N*-propionyldecanes (**14**, **15**) served as starting compounds also for the synthesis of derivatives **57**–**60**. The reaction of intermediates **14** and **15** with the commercial chloromethyl quinoline hydrochloride (**55**) or bromomethyl quinoxaline (**56**) [27] in the presence of K_2_CO_3_, furnished the desired derivatives (Scheme 4).

### 2.2. Radioligand Binding Assay

The newly synthesized compounds **20**–**27**, **53**–**54** and **57**–**60** were assayed in binding studies on μ-, δ-, and κ-opioid receptors (Table 1), performed on mouse brain homogenates in the presence of ^3^H-DAMGO for μ-receptor, ^3^H-DELTORPHINE II for δ-receptor and ^3^H-U69593 for κ-receptor. By comparison, the *K*_i_ value of the reference compound morphine is reported. Within the 9,10-diazatricyclo[4.2.1.1^2,5^]decane (**20**–**23**, **53**, **57** and **59**) and 2,7-diazatricyclo[4.4.0.0^3,8^]decane (**24**–**27**, **54**, **58** and **60**) series, μ-opioid receptor affinities fall within the 22 and >1000 nM range, whereas all compounds exhibited >1 μM *K*_i_ affinity values for δ- and κ-receptors. Compound **20** resulted to be the most interesting of the indenylidenic-9,10-diazatricyclo[4.2.1.1^2,5^]decane series with a μ-opioid receptor affinity of 50 nM. The introduction of a fluorine (**21**), chlorine (**22**) or bromine (**23**) atom on C5 of 2,3-dihydro-1*H*-indene system reveals a different impact on μ-receptor affinity. Only compound **21**, bearing the fluorine atom, maintained a receptor affinity (*K*_i_ = 65 nM) similar to **20**, whereas derivatives **22** (Cl) and **23** (Br) showed *K*_i_ values > 1000 nM. The substitution of the indenylidenic side chain with a heteroaromatic bicyclic system on DTD scaffold afforded compounds **53**, **57** and **59**. Within this small series only the indolic derivative **53**, 9-propionyl-10-[(1*H*-indol-2-yl)methyl]-9,10-diazatricyclo[4.2.1.1^2,5^]decane, showed an interesting μ-receptor affinity (*K*_i_ = 22 nM), resulting the best among all compounds reported herein. The replacement of the 9,10-diazatricyclo[4.2.1.1^2,5^]decane ring system with the 2,7-diazatricyclo[4.4.0.0^3,8^]decane unit resulted in derivatives **24**–**27**, **54**, **58** and **60**, which showed comparable effects on μ-binding affinity. Compound **24** showed a 2-fold lower affinity (*K*_i_ = 100 nM) if compared to its isomer **20**, whereas the introduction of a chlorine atom in the indene ring, compound **26**, led to a comparable μ-receptor affinity (*K*_i_ = 75 nM) with both **20** and **24**. The introduction of the indolic chain, compound **54**, resulted in a 23-fold lower affinity (*K*_i_ = 500 nM) with respect to its isomer **53**. In general, the introduction of a methylene-quinoline (**57**, **58**) or -quinoxaline (**59**, **60**) substituent on a nitrogen atom of DTD templates led to a decrease of μ-receptor affinity.

### 2.3. Molecular Docking

**Figure 5:**
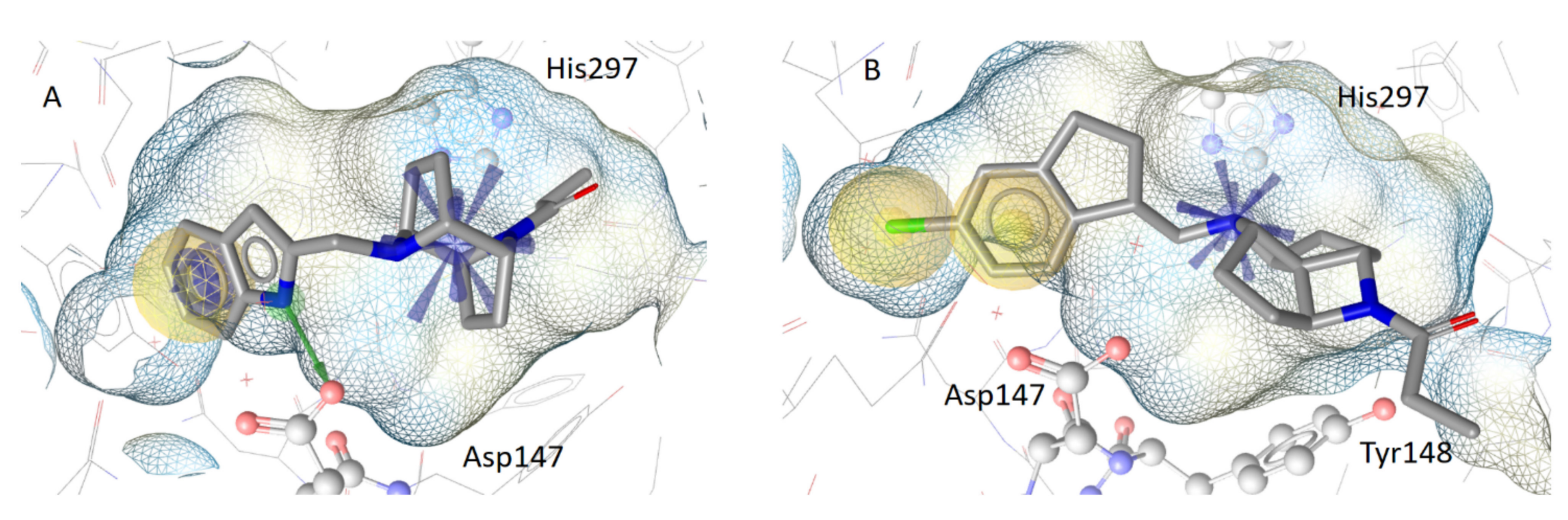
(**A**) Binding mode on the μ -opioid receptor of compound **53** (*K*_i_ = 22 nM), the most active compound of the 9,10-diazatricyclo[4.2.1.1^2,5^]decanes and the whole series. The nitrogen of the indole group acts as a hydrogen bond donor for Asp147. The tertiary nitrogen atoms of the 9,10-diazatricyclo[4.2.1.1^2,5^]decane form a positive ionic interaction with Asp147 and His297 on the other side of the binding pocket. (**B**) Binding mode of compound **26** on the μ-opioid receptor. One nitrogen of the 2,7-diazatricyclo[4.4.0.0^3,8^]decane system forms a positive ionic interaction with His297, Asp147 and Tyr 148.

**Figure 6:**
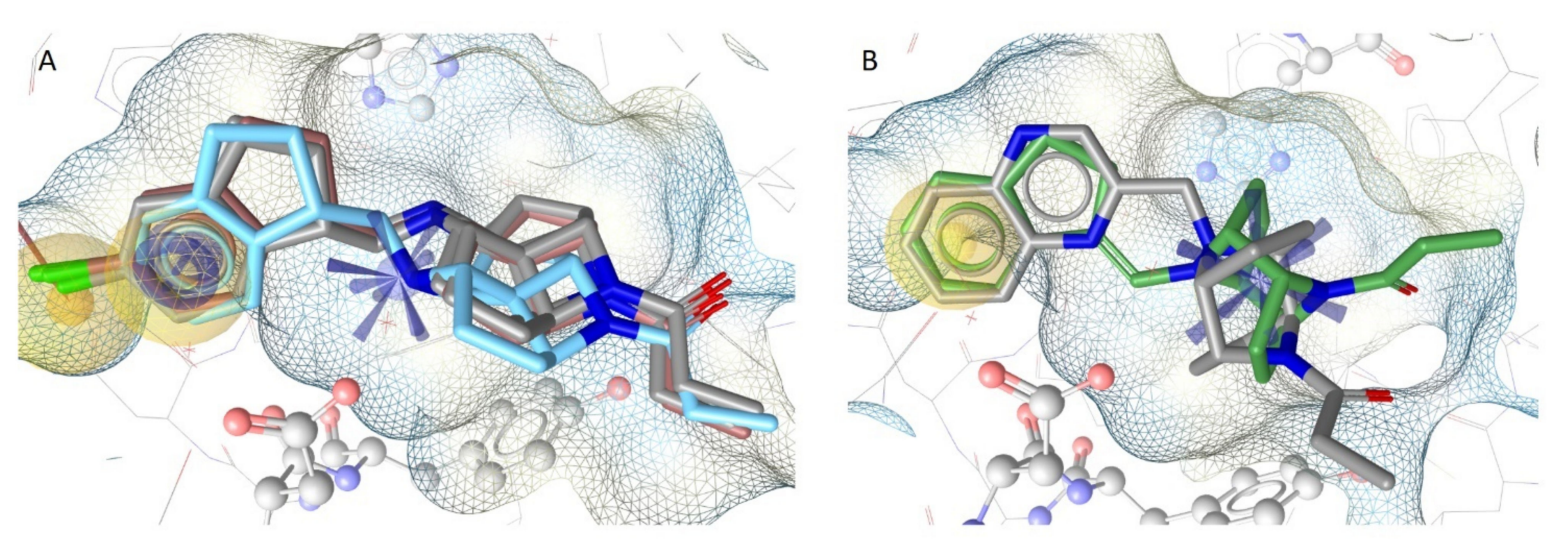
(**A**) Binding 2,7-diazatricyclo[4.4.0.0^3,8^]decanes **24**, **26**, **27** (grey) and nonbinding **25** in blue. (**B**) Compound **53** (*K*_i_ = 22 nM) in green and nonbinding compound **59** (grey).

The 9,10-diazatricyclo[4.2.1.1^2,5^]decane (**20**–**23**, **53**, **57** and **59**) and 2,7-diazatricyclo[4.4.0.0^3,8^]decane (**24**–**27**, **54**, **58** and **60**) series were docked into the binding site of the μ-opioid receptor. While the docking score assigned by the docking program to evaluate the pose quality did not correlate with the measured affinity, the activity seems to hinge around the positioning of the DTD ring. In the 9,10-diazatricyclo[4.2.1.1^2,5^]decanes, the ring is located and interacting with His297 and Asp147, while the DTD ring in the 2,7-diazatricyclo[4.4.0.0^3,8^]decanes is interacting also with Tyr148 (Figure 5). Slight shifts in the orientation of the ring, caused by the substitutions, seem to reduce ligand binding (Figure 6).

## 3. Materials and Methods

### 3.1. General Information

Melting points were uncorrected and were taken in open capillaries on a Köfler hot stage (Fisher Scientific, Landsmeer, NL) or Digital Electrothermal (Electrothermal, Stone, UK) melting point apparatus and are uncorrected. ^1^H-nuclear magnetic resonance (NMR) were determined in CDCl_3_ and were recorded with a an Avance III 400 NanoBay (Bruker, Billerica, MA, USA) or an XL-200 (200 MHz) (Varian, Palo Alto, CA, USA) instrument. Chemical shifts (*δ* scale) are reported in parts per million (ppm) downfield from tetramethylsilane (TMS) used as internal standard. The chemical shift values are reported in ppm (δ) and coupling constants (*J*) in Hertz (Hz). Signal multiplicities are represented by: s (singlet), d (doublet), dd (double doublet), t (triplet), q (quadruplet) and m (multiplet). The assignment of exchangeable protons (OH and NH) was confirmed by the addition of D_2_O. ^13^C-NMR spectra were determined in DMSO-d_6_ and were recorded at 100 MHz with the Bruker Avance III 400 NanoBay instrument. Mass spectra were recorded with a combined liquid chromatograph-1100 series Mass Selective Detector (MSD) system (Agilent, Santa Clara, CA, USA). Flash chromatography (FC) was performed using 70–230 mesh silica gel 60 (Merck, Kenilworth, NJ, USA). Light petroleum refers to the fraction with b.p. 40–60 °C. The progress of the reactions, the Rf and the purity of the final compounds were monitored by TLC using F-254 commercial plates (Merck). Elemental analysis was performed on a model 2400 instrument (Perkin-Elmer, Waltham, MA, USA) at Laboratorio di Microanalisi, Department of Chemistry and Pharmacy, University of Sassari, Italy, and the results were within ±0.4% of theoretical values.

### 3.2. Synthetic Methods

#### 3.2.1. Starting Materials, Intermediates and Known Compounds

Indanones **32**–**35**, 2-indolacetic acid and chloromethylquinoline hydrochloride (**55**) were purchased commercial products. The amine intermediates 9-propionyl-9,10-diazatricyclo[4.2.1.1^2,5^]decane (**14**), and 2-propionyl-2,7-diazatricyclo[4.4.0.0^3,8^]decane (**15**), the benzyl derivatives of 9,10-diazatricyclo[4.2.1.1^2,5^]decane (**44**) and 2,7-diazatricyclo[4.4.0.0^3,8^]decane were synthesized in accordance with [25] and bromomethyl quinoxaline (**56**) in accordance with [27].

#### 3.2.2. General Procedure for the Synthesis of Esters **36a**–**c**, **37a**–**c**, **38a**–**c** and **39a**–**c**

A suspension of triethyl phosphonacetate (TEFA, 0.93 mL, 4.7 mmol) and NaH (5.64 mmol, 60% suspension in mineral oil) in dry toluene (40 mL) was stirred at 4 °C for 1 h, under nitrogen atmosphere, then the mixture was brought to room temperature and the appropriate indanone (4.7 mmol) was added: the whole mixture was then refluxed at 110 °C for 20 h. After cooling, the mixture was washed with water and the solvent dried (Na_2_SO_4_) and evaporated under vacuum to obtain a mixture of *exo-E* (**36a**–**39a**), *exo-Z* (**36b**–**39b**) and *endo* (**36c**–**39c**) ester isomers, as previously reported for **36**–**39a**,**b** and **36c** [28]. Purification by flash chromatography, eluting with petroleum ether/ethyl ether 98/2 (**36a**,**b**,**c**; **37a**,**b**,**c**; **39a**,**b**,**c**) or petroleum ether/ethyl ether 95/5 (**38a**,**b**,**c**), afforded the pure *exo-Z*, *exo-E* and *endo* ester isomers.

#### 3.2.3. General Procedure for the Synthesis of Alcohols **40**–**43**

To a solution of esters **36a**–**39a** (540 mg, 0.228 mmol) in dry toluene (15.60 mL) under nitrogen atmosphere, a 25% solution of diisobutylaluminium hydride (DIBAL-H) in dry toluene (4.32 mL, 6.42 mmol) was added at 0 °C and the mixture stirred for 1 h. Then, a saturated K^+^/Na^+^ tartrate aqueous solution was added and the mixture was stirred overnight at room temperature. The mixture was taken up with diethyl ether and the organic phase was separated, washed (H_2_O), dried (Na_2_SO_4_) and evaporated under vacuum to afford the pure alcohols as an oil (**40**) [29] or a solid (**41**–**43**).

#### 3.2.4. General Procedure for the Synthesis of Aldehydes **16**–**19**

A solution of alcohol **40**–**43** (180 mg, 0.753 mmol) and MnO_2_ 85% (161 mg, 1.85 mmol) in CH_2_Cl_2_ was stirred at room temperature for 3 h. The unreacted MnO_2_ was filtered off and the solvent was evaporated under vacuum to give the pure aldehydes **16**–**19** as yellow solids.

#### 3.2.5. General Procedure for the Synthesis of Compounds **20**–**31**

A mixture of the appropriate 9-*N*-propionyl-10-diazatricyclo[4.2.1.1^2,5^]decane (**14**) [25] or 2-*N*-propionyl-7-diazatricyclo[4.4.0.0^3,8^]decane (**15**) (160 mg, 1.08 mmol) [25], the required aldehyde (**16**–**19**) (190 mg, 0.98 mmol), NaCNBH_3_ (87 mg, 1.39 mmol) and few drops of acetic acid was stirred at room temperature for 7 h. The solution was concentrated and the obtained residue was solubilized in NH_4_OH 1N (9 mL) and extracted with ethyl ether. The organic phase was dried (Na_2_SO_4_) and evaporated under reduced pressure: the resulting gummy crude product was purified by flash chromatography to give the desired compounds **20**–**31** as oils. The reaction of the amine **14** gave only one product (**20**–**23**), whereas the same reaction on amine **15** furnished both compounds **24**–**27** and **28**–**31** with similar R_f_. Flash chromatography separation, eluting with a mixture of dichloromethane/acetone 8/2, led to the isolation of *exo*-compounds **24**–**27** as first fraction and the unexpected *endo*-compounds **28**–**31**, as second. All final compounds were converted into hydrochloride salts.

#### 3.2.6. Procedure for the Synthesis of Compounds **47** and **48**

A mixture of **44** or **45** (3 mmol) in toluene (7 mL), acyl chloride (**46**) (3 mmol) and triethylamine (3 mmol), was stirred at room temperature for 3 h. Then, 4 mL of water was added and continued to stir for another 10 min. The reaction mothers were extracted with CH_2_Cl_2_, which by in vacuum evaporation supplied crude products such as light-yellow solids.

#### 3.2.7. Procedure for the Synthesis of Compounds **49** and **50**

To an ethanol solution (7 mL) of the appropriate derivatives **47** or **48** (1.9 mmol), 0.20 g of 10% Pd/C was added. The mixture was hydrogenated at 60 °C for 7 h, then the catalyst was removed by Celite^®^ filtration and the solvent evaporated to obtain the pure compounds **49** and **50** as white solids.

#### 3.2.8. Procedure for the Synthesis of Compounds **51** and **52**

A solution of the derivative **49** or **50** (1.5 mmol) in tetrahydrofuran (THF) (23 mL) was dripped into a solution in THF of LiAlH_4_ (6 mmol) previously cooled to 0 °C with ice. The mixture was stirred overnight at room temperature and then it was cooled to 0 °C and taken up with ethyl ether (10 mL) and water (1 mL). The white solid that formed was removed and the solution was concentrated, the oily residue was taken up with a 1:1 mixture of water and dichloromethane; the separated organic phase was dried (Na_2_SO_4_) and evaporated under vacuum to give compounds **51** and **52** as light-yellow solids.

#### 3.2.9. Procedure for the Synthesis of Final Compounds **53** and **54**

A solution of propionic anhydride (14 mmol) in dichloromethane (0.7 mL) was added to a solution of the appropriate derivative **51** or **52** (1.4 mmol) in dichloromethane (1.40 mL), cooled with ice. The mixture was stirred under reflux for 1 h. In the end, NaOH is added up to basic pH and extracted with dichloromethane. After evaporation of the organic phase, compounds **53** and **54** were obtained as white solids.

#### 3.2.10. Procedure for the Synthesis of Final Compounds **57**–**60**

Commercial chloromethylquinoline hydrochloride (**55**) or bromomethylquinoxaline (**56**) [27] (0.96 mmol) were added to 9-*N*-propionyl-10-diazatricyclo[4.2.1.1^2,5^]decane (**14**) or 2-*N*-propionyl-7-diazatricyclo [4.4.0.0^3,8^] decane (**15**) (0.96 mmol) respectively, in 9 mL of acetone and in the presence of K_2_CO_3_ (0.96 mmol). The mixture was stirred overnight at 60 °C. In the end, the salt was filtered off and the liquor mothers were evaporated to obtain derivatives **57**–**60** as crude products that were purified by flash chromatography.

### 3.3. Biology—Opioid Binding Assay

Ligand binding assays were determined for compounds under study, at μ-, δ-, and κ-opioid receptors, as described in detail elsewhere [30,31]. Binding affinities for μ-, δ-, and κ-opioid receptors were determined by displacing, respectively, [^3^H]DAMGO (1 nM), [^3^H]DADLE (1 nM) and [^3^H]U69593 (1 nM) from mouse brain membrane binding sites. Brain membranes were incubated with the appropriate [^3^H]-ligand in 50 mM Tris–HCl buffer, pH 7.4, at 25 °C for 60 min in the absence or presence of 10 μM naloxone. IC_50_ values were determined from log dose displacement curves, and *K*_i_ values were calculated from the obtained IC_50_ values by means of the equation of Cheng and Prusoff [32].

### 3.4. Molecular Docking

Docking simulations were conducted with GOLD version 5.2 (The Cambridge Crystallographic Data Centre, Cambridge, UK). This program uses a genetic algorithm to calculate up to ten docking poses per input-ligand. The resulting poses were evaluated with the scoring function GoldScore that takes into account hydrogen bonding, ligand internal strains, and steric aspects of the receptor-ligand complex. The crystal structure of the β-FNA-MOR complex (PDB-entry 4DKL1) [33] was prepared for docking by adding hydrogens and deleting all water molecules except 718 and 719. The remaining two water molecules were set to “toggle and spin”. This allowed the program to automatically decide to include the water molecule in a simulation run and to optimize the orientation of the water molecule. The area of 6 Å around the co-crystallized ligand was defined as the binding site.

### 3.5. Experimental

Compounds **36a**, **36b**, **36c**, **39a** and **40** which were already published [28,29,34] are also characterized here.

*(E)-Ethyl 2-(2,3-dihydro-1H-inden-1-ylidene)acetate* (36a) [28]

Title compound was obtained in 9% of total yield; IR ν_max_ (film) cm^−1^: 1713. ^1^H-NMR (CDCl_3_): 7.62–7.58 (d, 1H, *J* = 7.6 Hz), 7.40–7.23 (m, 3H), 6.33–6.31 (m, 1H), 4.23 (q, 2H, *J* = 7.0 Hz), 3.35–3.27 (m, 2H), 3.11–3.05 (m, 2H), 1.33 (t, 3H, *J* = 7.0 Hz).

*(Z)-Ethyl 2-(2,3-dihydro-1H-inden-1-ylidene)acetate* (36b) [28]

Title compound was obtained in 7% of total yield; IR ν_max_ (film) cm^−1^: 1704. ^1^H-NMR (CDCl_3_): 8.82 (d, 1H, *J* = 7.4 Hz), 7.31–7.27 (m, 3H), 5.97 (s, 1H), 4.22 (q, 2H, *J* = 7.2 Hz), 2.95 (d, 4H, *J* = 5.2 Hz), 1.32 (t, 3H, *J* = 7.2 Hz).

*Ethyl 2-(1H-inden-3-yl)acetate* (36c) [28]

Title compound was obtained in 17% of total yield; IR ν_max_ (film) cm^−1^: 1739. ^1^H-NMR (CDCl_3_): 7.46 (d, 1H, *J* = 7.0 Hz), 7.42–7.21 (m, 3H), 6.44–6.40 (m, 1H), 4.17 (q, 2H, *J* = 7.2 Hz), 3.59 (s, 2H), 3.39 (s, 2H), 1.26 (t, 3H, *J* = 7.2 Hz).

*(E)-Ethyl 2-(5-fluoro-2,3-dihydro-1H-inden-1-ylidene)acetate* (37a)

Title compound was obtained in 20% of total yield; m.p. 75–77 °C. IR ν_max_ (nu*J*ol) cm^−1^: 1695. ^1^H-NMR (CDCl_3_): 7.55 (dd, 1H, *J*_m_ = 5.2 Hz, *J*_o_ = 8.4 Hz), 7.10–6.90 (m, 2H), 6.24 6.23 (m, 1H), 4.23 (q, 2H, *J* = 7.2 Hz), 3.38–3.29 (m, 2H), 3.12–3.04 (m, 2H), 1.35 (t, 3H, *J* = 7.2 Hz).

*(Z)-Ethyl 2-(5-fluoro-2,3-dihydro-1H-inden-1-ylidene)acetate* (37b)

Title compound was obtained in 14% of total yield; m.p. 30–39 °C. IR ν_max_ (nujol) cm^−1^: 1714. ^1^H-NMR (CDCl_3_): 8.95–8.80 (m, 1H), 7.02–6.90 (m, 3H), 5.93 (s, 1H), 4.20 (q, 2H, *J* = 7.2 Hz), 2.96 (s, 4H), 1.30 (t, 3H, *J* = 7.2 Hz).

*Ethyl 2-(6-fluoro-1H-inden-3-yl)acetate* (37c)

Title compound was obtained in 24% of total yield; IR ν_max_ (film) cm^−1^: 1737. ^1^H-NMR (CDCl_3_): 7.31–7.24 (m, 1H), 7.18–6.95 (m, 2H), 6.40 (s, 1H), 4.20 (q, 2H, *J* = 7.0 Hz), 3.56 (s, 2H), 3.36 (s, 2H), 1.26 (t, 3H, *J* = 7.0 Hz).

*(E)-Ethyl 2-(5-chloro-2,3-dihydro-1H-inden-1-ylidene)acetate* (38a)

Title compound was obtained in 18% of total yield; m.p. 46–47 °C. IR ν_max_ (nujol) cm^−1^: 1697. ^1^H-NMR (CDCl_3_): 7.53 (d, 1H, *J* = 8.2 Hz), 7.34 (s, 1H), 7.23 (dd, 1H, *J*_o_ = 8.4 Hz, *J*_m_ = 1.6 Hz), 6.28–6.25 (m, 1H), 4.22 (q, 2H, *J* = 7.2 Hz), 3.35–3.28 (m, 2H), 3.09–3.03 (m, 2H), 1.33 (t, 3H, *J* = 7.2 Hz).

*(Z)-Ethyl 2-(5-chloro-2,3-dihydro-1H-inden-1-ylidene)acetate* (38b)

Title compound was obtained in 10% of total yield; m.p. 45–47 °C. IR ν_max_ (nujol) cm^−1^: 1717. ^1^H-NMR (CDCl_3_): 8.80 (d, 1H, *J* = 8.0 Hz), 7.28–7.22 (m, 2H), 5.96 (s, 1H), 4.20 (q, 2H, *J* = 7.2 Hz), 2.95 (s, 4H), 1.31 (t, 3H, *J* = 7.2 Hz).

*Ethyl 2-(6-chloro-1H-inden-3-yl)acetate* (38c)

Title compound was obtained in 12% of total yield; IR ν_max_ (film) cm^−1^: 1734. ^1^H-NMR (CDCl_3_): 7.42 (s, 1H), 7.28–7.24 (d, 2H), 6.44 (s, 1H), 4.17 (q, 2H, *J* = 7.2 Hz), 3.57 (s, 2H), 3.37 (s, 2H), 1.26 (t, 3H, *J* = 7.2 Hz).

*(E)-Ethyl 2-(5-bromo-2,3-dihydro-1H-inden-1-ylidene)acetate* (39a) [34]

Title compound was obtained in 17% of total yield; m.p. 87–89 °C. IR ν_max_ (nujol) cm^−1^: 1696. ^1^H-NMR (CDCl_3_): 7.49 (d, 1H, *J* = 7.8 Hz), 7.45–7.32 (m, 2H), 6.30–6.27 (m, 1H), 4.22 (q, 2H, *J* = 7.2 Hz), 3.37–3.25 (m, 2H), 3.10–3.03 (m, 2H), 1.32 (t, 3H, *J* = 7.2 Hz).

*(Z)-Ethyl 2-(5-bromo-2,3-dihydro-1H-inden-1-ylidene)acetate* (39b)

Title compound was obtained in 13% of total yield; m.p. 48–49 °C. IR ν_max_ (nujol) cm^−1^: 1716. ^1^H-NMR (CDCl_3_): 8.72 (d, 1H, *J* = 8.6 Hz), 7.45 (s, 1H), 7.30 (dd, 1H, *J*_o_ = 8.6 Hz, *J*_m_ = 2.0 Hz,), 6.00–5.96 (m, 1H), 4.19 (q, 2H, *J* = 7.2 Hz), 2.94 (s, 4H), 1.31 (t, 3H, *J* = 7.2 Hz).

*Ethyl 2-(6-bromo-1H-inden-3-yl)acetate* (39c)

Title compound was obtained in 21% of total yield; IR ν_max_ (film) cm^−1^: 1730. ^1^H-NMR (CDCl_3_): 7.58 (s, 1H), 7.43 (d, 1H, *J* = 8.2 Hz), 7.24 (dd, 1H, *J*_o_ = 8.0 Hz, *J*_m_ = 2.2 Hz), 6.42–6.40 (m, 1H), 4.17 (q, 2H, *J* = 7.0 Hz), 3.56 (s, 2H), 3.37 (s, 2H), 1.26 (t, 3H, *J* = 7.0 Hz).

*(E)-2-(2,3-Dihydro-1H-inden-1-ylidene)ethanol* (40) [29]

Title compound was obtained in 92% of total yield; IR ν_max_ (film) cm^−1^: 3420. ^1^HNMR (CDCl_3_): 7.52–7.46 (m, 1H), 7.24–7.18 (m, 3H), 6.15–6.09 (m, 1H), 4.35 (d, 2H, *J* = 7.0 Hz), 3.05–2.99 (m, 2H), 2.83–2.75 (m, 2H).

*(E)-2-(5-fluoro-2,3-dihydro-1H-inden-1-ylidene)ethanol* (41)

Title compound was obtained in 47% of total yield; m.p. 50–53 °C; IR ν_max_ (nujol) cm^−1^: 3415. ^1^H-NMR (CDCl_3_): 7.40 (dd, 1H, *J*_o_ = 8.2 Hz, *J*_m_ = 5.2 Hz), 6.95–6.86 (m, 2H), 6.07–5.99 (m, 1H), 4.33 (d, 2H, *J* = 6.8 Hz), 3.00–2.97 (m, 2H), 2.85–2.77 (m, 2H).

*(E)-2-(5-chloro-2,3-dihydro-1H-inden-1-ylidene)ethanol* (42)

Title compound was obtained in 86% of total yield; m.p. 92–95 °C; IR ν_max_ (nujol) cm^−1^: 3203. ^1^H-NMR (CDCl_3_): 7.38 (dd, 1H, *J* = 8.2 Hz), 7.23 (s, 1H), 7.17 (dd, 1H, *J*_o_ = 8.0 Hz, *J*_m_ = 1.8 Hz), 6.18–6.00 (m, 1H), 4.33 (d, 2H, *J* = 6.8 Hz), 3.02–2.95 (m, 2H), 2.85–2.76 (m, 2H).

*(E)-2-(5-bromo-2,3-dihydro-1H-inden-1-ylidene)ethanol* (43)

Title compound was obtained in 33% of total yield; m.p. 82–84 °C; IR ν_max_ (nujol) cm^−1^: 3234. ^1^H-NMR (CDCl_3_): 7.40 (dd, 1H, *J* = 7.8 Hz), 7.32–7.20 (m, 2H), 6.15–6.04 (m, 1H), 4.33 (d, 2H, *J* = 6.8 Hz), 3.05–2.92 (m, 2H), 2.85–2.74 (m, 2H).

*(E)-2-(2,3-Dihydro-1H-inden-1-ylidene)acetaldehyde* (16)

Title compound was obtained in 63% of total yield; m.p. 69–70 °C; IR ν_max_ (nujol) cm^−1^: 1652. ^1^H-NMR (CDCl_3_): 10.05 (d, 1H, *J* = 7.8 Hz), 7.64 (d, 1H, *J* = 7.8 Hz), 7.43–7.22 (m, 3H), 6.54–6.48 (m, 1H), 3.35–3.29 (m, 2H), 3.21–3.13 (m, 2H).

*(E)-2-(5-Fluoro-2,3-dihydro-1H-inden-1-ylidene)acetaldehyde* (17)

Title compound was obtained in 67% of total yield; m.p. 100–103 °C; IR ν_max_ (nujol) cm^−1^: 1646. ^1^H-NMR (CDCl_3_): 10.02 (d, 1H, *J* = 7.6 Hz), 7.60 (dd, 1H, *J*_o_ = 8.6 Hz, *J*_m_ = 5.4 Hz), 7.08–6.96 (m, 2H), 6.46–6.40 (m, 1H), 3.38–3.30 (m, 2H), 3.19–3.12 (m, 2H).

*(E)-2-(5-Chloro-2,3-dihydro-1H-inden-1-ylidene)acetaldehyde* (18)

Title compound was obtained in 64% of total yield; m.p. 192–194 °C; IR ν_max_ (nujol) cm^−1^: 1657. ^1^H-NMR (CDCl_3_): 10.03 (d, 1H, *J* = 7.6 Hz), 7.55 (dd, 1H, *J*_o_ = 8.0 Hz, *J*_m_ = 5.0 Hz), 7.37 (s, 1H), 7.28 (dd, 1H, *J*_o_ = 8.4 Hz, *J*_m_ = 1.6 Hz), 6.49–6.43 (m, 1H), 3.37–3.30 (m, 2H), 3.18–3.11 (m, 2H).

*(E)-2-(5-Bromo-2,3-dihydro-1H-inden-1-ylidene)acetaldehyde* (19)

Title compound was obtained in 22% of total yield; m.p. 101–103 °C; IR ν_max_ (nujol) cm^−1^: 1655. ^1^H-NMR (CDCl_3_): 10.03 (d, 1H, *J* = 7.6 Hz), 7.55–7.38 (m, 3H), 6.50–6.46 (m, 1H), 3.38–3.29 (m, 2H), 3.20–3.12 (m, 2H).

*9-Propionyl-10-[(E)-1-(2-(2,3-dihydro-1H-inden-1-ylidene)ethyl]-9,10-diazatricyclo[4.2.1.1^2,5^]decane* (20)

Title compound was obtained in 64% of total yield; m.p. 132–134 °C (as hydrochloride); purified by FC (petroleum ether/ethyl acetate 6/4), IR ν_max_ (nujol) cm^−1^: 1633. ^1^H-NMR (CDCl_3_): 7.54–7.42 (m, 1H), 7.25–7.19 (m, 3H), 6.00–5.94 (m, 1H), 4.48 (s, 1H), 3.86 (s, 1H), 3.14–2.92 (m, 6H), 2.67–2.62 (m, 2H), 2.29 (q, 2H, *J* = 7.2 Hz), 2.11–1.54 (m, 8H), 1.16 (t, 3H, *J* = 7.2 Hz).). ^13^C-NMR (DMSO, 100 MHz) δ: 169.71 (CO), 150.16 (C), 142.64 (C), 138.55 (C), 129.91 (CH), 128.71 (CH), 124.47 (CH), 124.20 (CH), 111.30 (CH), 59.15 (2CH), 57.08 (CH), 53.84 (CH), 50,19 (CH_2_), 30.30 (CH_2_), 28.76 (CH_2_), 27.00 (CH_2_), 24.80 (2CH_2_), 23.48 (2CH_2_), 9.09 (CH_3_). LC/MS: *m*/*z* 337 [M + 1]. Elem. Anal. Calcd for C_22_H_28_N_2_O: C, 78.53; H, 8.39; N, 8.33. Found: C, 78.55; H, 8.14; N, 8. 28.

*9-Propionyl-10-[(E)-1-(2-(5-fluoro-2,3-dihydro-1H-inden-1-ylidene)ethyl]-9,10-diazatricyclo[4.2.1.1^2,5^]decane* (21)

Title compound was obtained in 72% of total yield; m.p. 181–183 °C (as hydrochloride); purified by FC (petroleum ether/ethyl acetate 3/7), IR ν_max_ (nujol) cm^−1^: 1633. ^1^H-NMR (CDCl_3_): 7.40 (d, 1H, *J* = 8.4 Hz), 7.22 (s, 1H), 7.18 (dd, 1H, *J*_o_ = 8.4 Hz, *J*_m_ = 1.6 Hz), 5.86–5.82 (m, 1H), 4.56–4.32 (m, 1H), 3.93–3.80 (m, 1H), 3.12–2.90 (m, 6H), 2.78–2.60 (m, 2H), 2.28 (q, 2H, *J* = 7.4 Hz), 2.14–1.48 (m, 8H), 1.16 (t, 3H, *J* = 7.4 Hz). ^13^C-NMR (DMSO, 100 MHz) δ: 169.71 (CO), 160.82 (C), 146.16 (C), 140.64 (C), 138.55 (C), 129.91 (CH), 120.47 (CH), 112.85 (CH), 111.30 (CH), 63.98 (2CH), 59.25 (CH), 54.65 (CH), 50,19 (CH_2_), 29.30 (CH_2_), 27.46 (CH_2_), 26.98 (CH_2_), 24.90 (2CH_2_), 23.68 (2CH_2_), 9.11 (CH_3_). LC/MS: *m*/*z* 355 [M + 1]. Elem. Anal. Calcd for C_22_H_27_FN_2_O: C, 74.55, H, 7.68, N, 7.90 Found. C,74.21, H, 7.54, N, 8.00.

*9-Propionyl-10-[(E)-1-(2-(5-chloro-2,3-dihydro-1H-inden-1-ylidene)ethyl]-9,10-diazatricyclo[4.2.1.1^2,5^]decane* (22)

Title compound was obtained in 47% of total yield; m.p. 170–173 °C (as hydrochloride); purified by FC (petroleum ether/ethyl acetate 6/4), IR ν_max_ (nujol) cm^−1^: 1633. ^1^H-NMR (CDCl_3_): 7.40 (d, 1H, *J* = 8.4), 7.22 (s, 1H), 7.18 (dd, 1H, *J*_o_ = 8.4 Hz, *J*_m_ = 1.6 Hz), 5.86–5.81 (m, 1H), 4.56–4.32 (m, 1H), 3.93–3.80 (m, 1H), 3.12–2.90 (m, 6H), 2.78–2.60 (m, 2H), 2.28 (q, 2H, *J* = 7.2 Hz), 2.14–1.48 (m, 8H), 1.16 (t, 3H *J* = 7.2 Hz). ^13^C-NMR (DMSO, 100 MHz) δ: 169.87 (CO), 148.82 (C), 145.97 (C), 138.88 (C), 133.32 (C), 126.82 (CH), 125.38 (CH), 122.39 (CH), 108.87 (CH), 65.97 (2CH), 59.25 (CH), 54.87 (CH), 51,33 (CH_2_), 29.34 (CH_2_), 27.76 (CH_2_), 26.87 (CH_2_), 24.92 (2CH_2_), 23.56 (2CH_2_), 10.12 (CH_3_). LC/MS: *m*/*z* 373 [M + 1], 371 [M + 1]. Elem Anal.: Calcd for C_22_H_27_ClN_2_O: C, 71.24, H, 7.34, N, 7.55 Found. C,71.21, H, 7.64, N, 7.70.

*9-Propionyl-10-[(E)-1-(2-(5-bromo-2,3-dihydro-1H-inden-1-ylidene)ethyl]-9,10-diazatricyclo[4.2.1.1^2,5^]decane* (23)

Title compound was obtained in 56% of total yield; m.p. 167–170 °C (as hydrochloride); purified by FC (petroleum ether/ethyl acetate 6/4), IR ν_max_ (nujol) cm^−1^: 1633. ^1^H-NMR (CDCl_3_): 7.40–7.16 (m, 3H), 6.01–5.93 (m, 1H), 4.54–4.42 (m, 1H), 3.93–3.80 (m, 1H), 3.11–2.89 (m, 6H), 2.72–2.67 (m, 2H), 2.28 (q, 2H, *J* = 7.0 Hz), 2.10–1.45 (m, 8H), 1.19 (t, 3H, *J* = 7.0 Hz). ^13^C-NMR (DMSO, 100 MHz) δ: 169.87 (CO), 149.15 (C), 146.36 (C), 139.24 (C), 129.61 (CH), 128.36 (CH), 122.70 (CH), 121.99 (C), 108.98 (CH), 65.98 (2CH), 59.25 (CH), 54.77 (CH), 51,33 (CH_2_), 29.32 (CH_2_), 27.67 (CH_2_), 26.98 (CH_2_), 24.92 (2CH_2_), 23.66 (2CH_2_), 10.12 (CH_3_). LC/MS: *m*/*z* 416 [M + 1]. Elem Anal.: Calcd for C_22_H_27_BrN_2_O: C, 63.61, H, 6.55, N, 6.74 Found. C, 63.29, H, 6.64, N, 7.00.

*2-Propionyl-7-[(E)-1-(2-(2,3-dihydro-1H-inden-1-ylidene)ethyl]-2,7-diazatricyclo[4.2.0.0^3,8^]decane* (24)

Title compound was obtained in 40% of total yield; m.p.167–170 °C (as hydrochloride); purified by FC (dichloromethane/acetone 8/2), IR ν_max_ (nujol) cm^−1^: 1633. ^1^H-NMR (CDCl_3_): 7.52–7.46 (m, 1H), 7.24–7.16 (m, 3H), 6.00–5.95 (m, 1H), 4.38–4.24 (m, 1H), 3.82–3.74 (m, 1H) 3.58–3.53 (m, 2H), 3.07–2.99 (m, 4H), 2.79–2.76 (m, 2H), 2.27 (q, 2H, *J* = 7.2 Hz), 2.00–1.25 (m, 8H), 1.16 (t, 3H, *J* = 7.2 Hz). ^13^C-NMR (DMSO, 100 MHz) δ: 170.71 (CO), 150.26 (C), 142.55 (C), 138.55 (C), 129.82 (CH), 128.65 (CH), 124.47 (CH), 124.20 (CH), 111.30 (CH), 59.26 (2CH), 57.08 (CH), 53.84 (CH), 50,19 (CH_2_), 30.30 (CH_2_), 28.76 (CH_2_), 27.00 (CH_2_), 24.80 (2CH_2_), 23.50 (2CH_2_), 9.11 (CH_3_). LC/MS: *m*/*z* 337 [M + 1]. Elem Anal.: Calcd. for C_22_H_28_N_2_O: C, 78.53, H, 8.39, N, 8.33 Found. C, 78.35, H, 8.64, N, 8. 38.

*2-Propionyl-7-[(E)-1-(2-(5-fluoro-2,3-dihydro-1H-inden-1-ylidene)ethyl]-2,7-diazatricyclo[4.2.0.0^3,8^]decane* (25)

Title compound was obtained in 35% of total yield; m.p. 131–133 °C (as hydrochloride); purified by FC (dichloromethane/acetone 8/2), IR ν_max_ (nujol) cm^−1^: 1633. ^1^H-NMR (CDCl_3_): 7.39 (dd, 1H, *J*_mH-F_ = 5.2 Hz, *J*_oH-H_ = 8.2, Hz), 6.93–6.81 (m, 2H), 5.90–5.80 (m, 1H), 4.35–4.27 (m, 1H), 3.86–3.82 (m, 1H) 3.56–3.49 (m, 2H), 3.06–2.96 (m, 4H), 2.80–2.77 (m, 2H), 2.26 (q, 2H, *J* = 7.0 Hz), 2.00–1.21 (8H, m, 4CH_2_), 1.16 (t, 3H, *J* = 7.0 Hz). ^13^C-NMR (DMSO, 100 MHz) δ: 172.22 (CO), 160.86 (C), 146.24 (C), 140.64 (C), 139.00 (C), 129.91 (CH), 120.48 (CH), 112.75 (CH), 111.30 (CH), 64.00 (2CH), 59.25 (CH), 54.65 (CH), 50,25 (CH_2_), 29.30 (CH_2_), 27.46 (CH_2_), 26.98 (CH_2_), 24.92 (2CH_2_), 23.68 (2CH_2_), 9.11 (CH_3_). LC/MS: *m*/*z* 355 [M + 1]. Elem. Anal. Calcd. for C_22_H_27_FN_2_O: C,74.55, H, 7.68, N, 7.90 Found. C, 74.33, H, 7.76, N, 8.10.

*2-Propionyl-7-[(E)-1-(2-(5-chloro-2,3-dihydro-1H-inden-1-ylidene)ethyl]-2,7-diazatricyclo[4.2.0.0^3,8^]decane* (26)

Title compound was obtained in 41% of total yield; m.p. 179–181 °C (as hydrochloride); purified by FC (dichloromethane/acetone 8/2), IR ν_max_ (nujol) cm^−1^: 1633. ^1^H-NMR (CDCl_3_): 7.30 (d, 1H, *J* = 8.2 Hz), 7.12 (s, 1H), 7.04 (d, 1H, *J* = 8.2 Hz), 5.90–5.76 (m, 1H), 4.28–4.18 (m, 1H), 3.72–3.65 (m, 1H) 3.48–3.18 (m, 2H), 3.02–2.81 (m, 4H), 2.72–2.60 (m, 2H), 2.17 (q, 2H, *J* = 7.2 Hz), 1.98–1.15 (m, 8H), 1.08 (t, 3H, *J* = 7.2 Hz). ^13^C-NMR (DMSO, 100 MHz) δ: 170.58 (CO), 149.43 (C), 148.73 (C), 139.27 (C), 133.84 (C), 127.35 (CH), 125.90 (CH), 122.76 (CH), 109.62 (CH), 57.97 (2CH), 57.32 (2CH), 51,39 (CH_2_), 29.82 (CH_2_), 28.31 (CH_2_), 25.10 (2CH_2_), 22.62 (2CH_2_), 22.16 (CH_2_), 10.12 (CH_3_). LC/MS: *m*/*z* 373 [M + 1]. 371 [M + 1]. Elem. Anal.: Calcd. for C_22_H_27_ClN_2_O: C, 71.24, H, 7.34, N, 7.55 Found. C, 71.46, H, 7.52, N, 7.30.

*2-Propionyl-7-[(E)-1-(2-(5-bromo-2,3-dihydro-1H-inden-1-ylidene)ethyl]-2,7-diazatricyclo[4.2.0.0^3,8^]decane* (27)

Title compound was obtained in 39% of total yield; m.p. 177–179 °C (as hydrochloride); purified by FC (dichloromethane/acetone 8/2), IR ν_max_ (nujol) cm^−1^: 1633. ^1^H-NMR (CDCl_3_): 7.29 (s, 1H), 7.22 (s, 1H), 5.90–5.78 (m, 1H), 4.25–4.14 (m, 1H), 3.73–3.65 (m, 1H) 3.45–3.43 (m, 2H), 2.98–2.87 (m, 4H), 2.70–2.67 (m, 2H), 2.18 (q, 2H, *J* = 7.2 Hz), 1.92–1.17 (m, 8H), 1.08 (3H, t, *J* = 7.2 Hz).). ^13^C-NMR (DMSO, 100 MHz) δ: 170.47 (CO), 149.15 (C), 146.40 (C), 139.25 (C), 129.61 (CH), 128.40 (CH), 122.70 (CH), 122.00 (C), 108.98 (CH), 65.98 (2CH), 59.25 (CH), 54.77 (CH), 51,33 (CH_2_), 29.32 (CH_2_), 27.67 (CH_2_), 26.98 (CH_2_), 24.92 (2CH_2_), 23.70 (2CH_2_), 10.14 (CH_3_). LC/MS: *m*/*z* 416 [M + 1]. Elem. Anal. Calcd. for C_22_H_27_BrN_2_O: C, 63.61, H, 6.55, N, 6.74 Found. C, 63.85, H, 6.70, N, 7.00.

*2-Propionyl-7-[(2-(1H-inden-3-yl)ethyl]-2,7-diazatricyclo[4.2.0.0^3,8^]decane* (28)

Title compound was obtained in 24% of total yield; m.p. 167–170 °C (as hydrochloride); purified by FC (dichloromethane/acetone 8/2), IR ν_max_ (nujol) cm^−1^: 1633. ^1^H-NMR (CDCl_3_): 7.50–7.17 (m, 4H), 6.27 (s, 1H), 4.31 (s, 1H), 3.77 (s, 1H), 3.34 (s, 2H) 3.08–3.00 (m, 4H), 2.72–2.64 (m, 2H), 2.26 (q, 2H, *J* = 7.0 Hz), 1.96–1.25 (m, 8H), 1.16 (t, 3H, *J* = 7.0 Hz). LC/MS: *m*/*z* 337 [M + 1]. Elem. Anal. Calcd. for C_22_H_28_N_2_O: C, 78.53, H, 8.39, N, 8.33 Found. C, 78.65, H, 8.52, N, 8. 34.

*2-Propionyl-7-[(2-(6-fluoro-1H-inden-3-yl)ethyl]-2,7-diazatricyclo[4.2.0.0^3,8^]decane* (29)

Title compound was obtained in 23% of total yield; m.p. 125–128 °C (as hydrochloride); purified by FC (dichloromethane/acetone 8/2), IR ν_max_ (nujol) cm^−1^: 1633. ^1^H-NMR (CDCl_3_): 7.25 (dd, 1H, *J*_mH-F_ = 5.2 Hz, *J*_oH-H_ = 8.2 Hz), 7.30–6.77 (m, 2H), 6.22 (s, 1H), 4.30 (s, 1H), 3.77 (s, 1H) 3.31 (s, 2H), 3.06–2.99 (m, 4H) 2.67–2.58 (m, 2H), 2.26 (q, 2H, *J* = 7.2 Hz), 1.95–1.27 (m, 8H), 1.16 (t, 3H, *J* = 7.2 Hz). LC/MS: *m*/*z* 355 [M + 1]. Elem. Anal. Calcd. for C_22_H_27_FN_2_O: C, 74.55, H, 7.68, N, 7.90 Found. C, 74.20, H, 7.86, N, 8.15.

*2-Propionyl-7-[(2-(6-chloro-1H-inden-3-yl)ethyl]-2,7-diazatricyclo[4.2.0.0^3,8^]decane* (30)

Title compound was obtained in 22% of total yield; m.p. 143–146 °C (as hydrochloride); purified by FC (dichloromethane/acetone 8/2), IR ν_max_ (nujol) cm^−1^: 1633. ^1^H-NMR (CDCl_3_): 7.41 (s, 1H), 7.28–7.20 (m, 2H), 6.26 (s, 1H), 4.30 (s, 1H), 3.76 (s, 1H) 3.31 (s, 2H), 3.05–2.98 (m, 4H), 2.67–2.63 (m, 2H), 2.24 (q, 2H, *J* = 7.2 Hz), 1.95–1.32 (m, 8H), 1.16 (t, 3H, *J* = 7.2 Hz). LC/MS: *m*/*z* 372 [M + 1]. Elem. Anal. Calcd. for C_22_H_27_ClN_2_O: C, 71.24, H, 7.34, N, 7.55 Found C, 71.20, H, 7.52, N, 7.40.

*2-Propionyl-7-[(2-(6-bromo-1H-inden-3-yl)ethyl]-2,7-diazatricyclo[4.2.0.0^3,8^]decane* (31)

Title compound was obtained in 22% of total yield; m.p. 143–146 °C (as hydrochloride); purified by FC (dichloromethane/acetone 8/2), IR ν_max_ (nujol) cm^−1^: 1633. ^1^H-NMR (CDCl_3_): 7.58 (s, 1H), 7.42 (d, 1H, *J* = 8.0 Hz), 6.25 (s, 1H), 4.30 (s, 1H), 3.77 (s, 1H), 3.31 (s, 2H), 3.05–3.02 (m, 4H), 2.67–2.63 (m, 2H), 2.26 (q, 2H, *J* = 7.2 Hz), 2.00–1.26 (m, 8H), 1.16 (t, 3H, *J* = 7.2 Hz). LC/MS: *m*/*z* 416 [M + 1]. Elem. Anal. Calcd. C_22_H_27_BrN_2_O: C, 63.61, H, 6.55, N, 6.74 Found C, 63.70, H, 6.90, N, 6.88.

*(10-Benzyl-9, 10-diazatricyclo[4.2.1.1^2.5^]dec-9-yl)-(1H-indol-2-yl)-methanone* (47)

Title compound was obtained in 95% of total yield; m.p. 184 °C; purified by FC (petrol ether/ethyl acetate 7/3), IR ν_max_ (nujol) cm^−1^: 3273, 1607. ^1^H-NMR (CDCl_3_): 9.29 (s, 1H), 7.63 (d, *J* = 7.2 Hz, 1H), 7.45–7.05 (m, 8H), 6.78 (s, 1H), 4.77–4.62 (m, 2H), 3. (s, 2H), 3.15–3.00 (m, 2H), 2.30–1.20 (m, 8H). LC/MS: *m*/*z* 372 [M + 1]. Elem Anal.: Calcd for C_24_H_25_N_3_O: C, 77.60, H, 6.78, N, 11.31 Found. C, 77.29, H, 6.94, N, 11.00. 43.

*(7-Benzyl-2, 7-diazatricyclo[4.4.0.0^3.8^]dec-2-yl)-(1H-indol-2-yl)-methanone* (48)

Tilte compound was obtained in 25% of total yield; m.p. 218–220 °C; purified by FC (petrol ether/ethyl acetate 7/3), IR ν_max_ (nujol) cm^−1^: 3226, 1586. ^1^H-NMR (CDCl_3_): 9.34 (s, 1H), 7.66 (d, *J* = 8.0 Hz, 1H), 7.48–7.10 (m, 8H), 6.81 (s, 1H), 4.58–4.46 (m, 1H), 4.45–4.40 (m, 1H), 4.00 (d, *J* = 5.6 Hz, 2H), 3.07–3.05 (m, 1H), 3.04–2.97 (m, 1H), 2.35–1.45 (m, 8H). LC/MS: *m*/*z* 372 [M + 1]. Elem Anal.: Calcd for C_24_H_25_N_3_O: C, 77.60, H, 6.78, N, 11.31 Found. C, 77.22, H, 7.00, N, 11.60.

*(9,10-Diazatricyclo[4.2.1.1^2.5^]dec-9-yl)-(1H-indol-2-yl)-methanone* (49)

Title compound was obtained in 87% of total yield; m.p. 225 °C; IR ν_max_ (nujol) cm^−1^: 3323, 1596. ^1^H-NMR (CDCl_3_): 9.23 (s, 1H), 7.66 (d, *J* = 7.6 Hz, 1H), 7.43 (d, *J* = 7.8 Hz, 1H), 7.33–7.14 (m, 2H), 6.81 (s, 1H), 4.79–4.60 (m, 2H), 3.50–3.30 (m, 2H), 2.20–1.46 (m, 8H). LC/MS: *m*/*z* 282 [M + 1]. Elem Anal.: Calcd for C_17_H_19_N_3_O: C, 72.57, H, 6.81, N, 14.94 Found. C, 72.25, H, 6.94, N, 15.20.

*(2,7-Diazatricyclo[4.4.0.0^3.8^]dec-2-yl)-(1H-indol-2-yl)-methanone* (50)

Title compound was obtained in 88% of total yield; m.p. 240 °C; IR ν_max_ (nujol) cm^−1^: 3392, 1592. ^1^H-NMR (CDCl_3_): 9.45 (s, 1H), 7.66 (d, *J* = 7.8 Hz, 1H), 7.45 (d, *J* = 8.0 Hz, 1H), 7.33–7.10 (m, 2H), 6.81 (s, 1H), 4.50–4.42 (m, 1H), 4.41–4.34 (m, 1H), 3.40–3.30 (m, 1H), 3.28–3.20 (m, 1H), 2.42–1.43 (m, 8H). LC/MS: *m*/*z* 282 [M + 1]. Elem Anal.: Calcd for C_17_H_19_N_3_O: C, 72.57, H, 6.81, N, 14.94 Found. C, 72.40, H, 7.00, N, 15.00.

*9-(1H-Indol-2-yl methyl)-9, 10-diazatricyclo[4.2.1.1^2.5^]decane* (51)

Title compound was obtained in 93% of total yield; m.p. 109 °C; IR ν_max_ (nujol) cm^−1^: 3324, 1586. ^1^H-NMR (CDCl_3_): 8.54 (s, 1H), 7.54 (d, *J* = 7.4 Hz, 1H), 7.39 (d, *J* = 7.4 Hz, 1H), 7.20–7.00 (m, 2H), 6.29 (s, 1H), 3.80–3.65 (m, 1H), 3.48 (s, 2H), 3.18–3.06 (m, 1H), 2.95–2.80 (m, 2H), 2.10–1.50 (m, 8H). LC/MS: *m*/*z* 268 [M + 1]. Elem Anal.: Calcd for C_17_H_21_N_3_: C, 76.37, H, 7.92, N, 15.72 Found. C, 75.98, H, 8.34, N, 15.55.

*2-(1H-Indol-2-yl methyl)-2, 7-diazatricyclo[4.4.0.0^3.8^]decane* (52)

Title compound was obtained in 100% of total yield; m.p. 276 °C; IR ν_max_ (nujol) cm^−1^: 3350, 1596. ^1^H-NMR (CDCl_3_): 9.30 (s, 1H), 7.65 (d, *J* = 7.8 Hz, 1H), 7.44 (d, *J* = 7.8 Hz, 1H), 7.20–7.05 (m, 2H), 6.33 (s, 1H), 4.50–4.41 (m, 1H), 3.42–4.35 (m, 1H), 3.75 (s, 2H), 3.38–3.28 (m, 1H), 3.27–3.19 (m, 1H), 2.40–1.41 (m, 8H). LC/MS: *m*/*z* 268 [M + 1]. Elem Anal.: Calcd for C_17_H_21_N_3_: C, 76.37, H, 7.92, N, 15.72 Found. C, 76.00, H, 8.25, N, 15.98.

*9-Propionyl-10-(1H-indol-2-yl methyl)-9, 10-diazatricyclo[4.2.1.1^2.5^]decane* (53)

Title compound was obtained in 64% of total yield; m.p. 106 °C; purified by FC (dichloromethane/acetone 8/2), IR ν_max_ (nujol) cm^−1^: 3469, 1599. ^1^H-NMR (CDCl_3_): 8.42 (s, 1H), 7.55 (d, *J* = 7.4 Hz, 1H), 7.39 (d, *J* = 7.4 Hz, 1H), 7.30–7.05 (m, 2H), 6.33 (s, 1H), 4.55–4.45 (m, 1H), 3.91–3.84 (m, 1H), 3.54 (s, 2H), 3.10–2.95 (m, 2H), 2.49 (q, *J* = 7.6 Hz, 2H), 2.37–1.55 (m, 8H), 1.16 (t, *J* = 7.6 Hz, 3H). ^13^C-NMR (DMSO, 100 MHz) δ: 170.68 (CO), 136.78 (C), 135.52 (C), 133.00 (C), 130.99 (CH), 130.10 (CH), 129.34 (CH), 128.80 (CH), 128.72 (CH), 64.70 (2CH), 60.10 (CH), 57,12 (CH_2_), 28.58 (CH_2_), 27.55 (CH_2_), 25.20 (CH_2_), 24.68 (CH_2_), 24.14 (CH_2_), 9.60 (CH_3_). LC/MS: *m*/*z* 324 [M + 1]. Elem Anal.: Calcd for C_20_H_25_N_3_O: C, 74.27, H, 7.79, N, 12.99 Found. C, 74.65, H, 7.64, N, 13.20.

*2-Propionyl-7-(1H-indol-2-yl methyl)-2, 7-diazatricyclo[4.4.0.0^3.8^]decane* (54)

Title compound was obtained in 79% of total yield; m.p. 54 °C; purified by FC (chloroform/methanol 98/2); IR ν_max_ (nujol) cm^−1^: 3200, 1592. ^1^H-NMR (CDCl_3_): 9.40 (s, 1H), 7.67 (d, *J* = 8.0 Hz, 1H), 7.45 (d, *J* = 8.0 Hz, 1H), 7.40–7.10 (m, 2H), 6.82 (s, 1H), 4.75–4.50 (m, 4H), 4.12–3.91 (m, 2H), 2.38 (q, *J* = 7.6 Hz, 2H), 2.18–1.40 (m, 8H), 1.22 (t, *J* = 7.6 Hz, 3H). ^13^C-NMR (DMSO, 100 MHz) δ: 171.08 (CO), 136.76 (C), 135.52 (C), 133.00 (C), 131.00 (CH), 130.40 (CH), 129.34 (CH), 128.80 (CH), 128.66 (CH), 64.55 (2CH), 60.10 (CH), 57.32 (CH_2_), 28.58 (CH_2_), 27.55 (CH_2_), 25.20 (CH_2_), 24.68 (CH_2_), 24.14 (CH_2_), 9.60 (CH_3_). LC/MS: *m*/*z* 324 [M + 1]. Elem Anal.: Calcd for C_20_H_25_N_3_O: C, 74.27, H, 7.79, N, 12.99 Found. C, 74.54, H, 7.92, N, 13.35.

*9-Propionyl-10-(quinolyn-2-yl methyl)-9,10-diazatricyclo[4.2.1.1^2,5^]decane* (57)

Title compound was obtained pure in 57% of total yield; m.p. 59 °C; purified by FC (petroleum ether/ethyl acetate 6/4 and then only ethyl acetate); IR ν_max_ (nujol) cm^−1^: 1594. ^1^H-NMR (CDCl_3_): 8.18 (d, *J* = 8.0 Hz, 1H), 8.04 (d, *J* = 8.0 Hz, 1H), 7.87–7.79 (m, 2H), 7.75–7.65 (m, 1H), 7.56–7.45 (m, 1H), 4.55–4.45 (m,1H), 3.80–3.71 (m, 1H), 3.69 (s, 2H) 3.09–2.95 (m, 2H), 2.28 (q, *J* = 7.6 Hz, 2H), 2.14–1.55 (m, 8H) 1.15 (t, *J* = 7.6 Hz, 3H).). ^13^C-NMR (DMSO, 100 MHz) δ: 168.88 (CO), 155.52 (C), 145.57 (CH), 141.00 (C), 140.78 (C), 132,02 (CH), 130.10 (CH), 129.34 (CH), 128.80 (CH), 128.72 (CH), 64.70 (2CH), 60.10 (CH), 57,12 (CH_2_), 28.58 (CH_2_), 27.55 (CH_2_), 25.00 (CH_2_), 24.68 (CH_2_), 24.14 (CH_2_), 9.60 (CH_3_). LC/MS: *m*/*z* 348 [M + 1]. Elem Anal.: Calcd for C_22_H_25_N_3_O: C, 76.05; H, 7.25; N, 12.09; Found. C, 75.89, H, 7.44, N, 12.41.

*2-Propionyl-7-(quinolyn-2-yl methyl)-2,7-diazatricyclo[4.2.0.0^3,8^]decane* (58)

Title compound was obtained pure in 100% of total yield; m.p. 216 °C (as hydrochloride); IR ν_max_ (nujol) cm^−1^: 1613. ^1^H-NMR (CDCl_3_): 8.21 (d, *J* = 8.0 Hz, 1H), 8.04 (d, *J* = 8.0 Hz, 1H), 7.86–7.53 (m, 4H), 4.85 (s, 2H), 4.45–4.25 (m, 1H), 3.94–3.84 (m, 1H), 3.17–3.00 (m, 2H), 2.29 (q, *J* = 7.6 Hz, 2H), 2.18–1.30 (m, 8H) 1.18 (t, *J* = 7.6 Hz, 3H). ^13^C-NMR (DMSO, 100 MHz) δ: 170.60 (CO), 155.52 (C), 145.50 (CH), 141.20 (C), 140.80 (C), 132,02 (CH), 130.15 (CH), 129.32 (CH), 128.85 (CH), 128.72 (CH), 64.64 (2CH), 60.10 (CH), 57,10 (CH_2_), 28.80 (CH_2_), 27.55 (CH_2_), 25.00 (CH_2_), 24.66 (CH_2_), 24.15 (CH_2_), 9.50 (CH_3_). LC/MS: *m*/*z* 348 [M + 1]. Elem Anal.: Calcd for C_22_H_25_N_3_O: C, 76.05; H, 7.25; N, 12.09; Found. C, 75.89, H, 7.44, N, 12.41.

*9-Propionyl-10-(quinoxalyn-2-yl methyl)-9,10-diazatricyclo[4.2.1.1^2,5^]decane* (59)

Title compound was obtained in 100% of total yield; m.p. 128 °C; purified by FC (petroleum ether/ethyl acetate 7/3 and then only ethyl acetate), IR ν_max_ (nujol) cm^−1^: 1633. ^1^H-NMR (CDCl_3_): 9.25 (s, 1H), 8.01–8.14 (m, 2H), 7.74–7.79 (m, 2H), 4.45–4.55 (m, 1H), 3.81–3.94 (m, 1H), 3.75 (s, 2H), 3.00–3.15 (m, 2H), 2.29 (q, *J* = 8 Hz, 2H), 1.55–2.13 (m, 8H) 1.16 (t, 3H, *J* = 7.6 Hz). ^13^C-NMR (DMSO, 100 MHz) δ: 168.87 (CO), 155.50 (C), 145.57 (CH), 140.99 (C), 140.78 (C), 130.09 (CH), 129.54 (CH), 128.82 (CH), 128.70 (CH), 64.68 (2CH), 60.10 (CH), 57,14 (CH_2_), 28.58 (CH_2_), 27.54 (CH_2_), 25.04 (CH_2_), 24.68 (CH_2_), 24.14 (CH_2_), 9.50 (CH_3_). LC/MS: *m*/*z* 337 [M + 1]. Elem Anal.: Calcd for C_20_H_24_N_4_O: C, 71.40; H, 7.19; N, 16.65; Found. C, 71.29, H, 6.94, N, 17.00.

*2-Propionyl-7-(quinoxalyn-2-yl methyl)-2,7-diazatricyclo[4.4.0.0^3.8^]decane* (60)

Title compound was obtained in 5% of total yield; as pink-orange oil; purified by FC (petroleum ether/ethyl acetate 6/4, then only ethyl acetate), IR ν_max_ (nujol) cm^−1^: 1617. ^1^H-NMR (CDCl_3_): 9.12 (s, 1H), 8.15–8.00 (m, 2H), 7.85–7.70 (m, 2H), 4.40–4.35 (m, 1H), 4.29 (d, *J* = 6 Hz, 2H), 3.90–3.80 (m, 1H), 3.25–3.12 (m, 2H), 2.29 (q, *J* = 8 Hz, 2H), 1.55–2.13 (m, 8H) 1.17 (t, 3H, *J* = 7.6 Hz).). ^13^C-NMR (DMSO, 100 MHz) δ: 170.27 (CO), 155.50 (C), 145.57 (CH), 141.04 (C), 140.80 (C), 130.15 (CH), 129.74 (CH), 128.82 (CH), 128.70 (CH), 64.68 (2CH), 60.12 (CH), 57.14 (CH_2_), 28.58 (CH_2_), 27.54 (CH_2_), 25.06 (CH_2_), 24.70 (CH_2_), 24.14 (CH_2_), 9.50 (CH_3_). LC/MS: *m*/*z* 337 [M + 1]. Elem Anal.: Calcd for C_20_H_24_N_4_O: C, 71.40; H, 7.19; N, 16.65; Found. C, 71.69, H, 7.54, N, 17.00.

## 4. Conclusions

In summary, the purpose of this study was to better describe the impact of the cinnamyl side chain to enhance the binding with the opioid receptor site. This was achieved by the inclusion of a methyl group on the cinnamyl chain both into a rigid benzocondensed structure and into a bicyclic heteroaromatic system. Herein we have reported the synthesis of a small series of compounds containing 9-propionyl-10-substituted-9,10-diazatricyclo[4.2.1.1^2,5^]decane (compounds **20**–**23**, **53**, **57** and **59**), the 2-propionyl-7-substituted-2,7-diazatricyclo[4.4.0.0^3,8^]decane (compounds **24**–**27**, **54**, **58** and **60**) and the 2-propionyl-7-substituted-2,7-diazatricyclo[4.4.0.0^3,8^]decane (compounds **28**–**31**) cores.

Derivatives **20**–**27**, **53**, **54** and **57**–**60** were evaluated in μ-, δ- and κ-opioid receptor binding assays and, in general, both series showed higher μ-receptor selectivity than that of previously reported methylarylcinnamyl analogs. On the other hand, these novel ligands showed a reduced μ-affinity compared to the previous series.

From these studies, it is possible deduce that the incorporation of the methyl group on cinnamic chain into a rigid benzo-condensed structure led to templates endowed with 10-fold less affinity towards μ-receptors and negligible for δ- and κ-receptors with >1 μM *K*_i_ affinity values, but at the same time the resulting indenylidenic group is responsible of increased μ-receptor selectivity. Compound **20** turned out as the most promising derivative from the indenylidenic-9,10-diazatricyclo[4.2.1.12,5]decane series with a μ-opioid receptor affinity of 50 nM. The substitution with a fluorine (**21**), chlorine (**22**) or bromine atom (**23**) on C5 of 2,3-dihydro-1H-indene system uncovers a different effect on μ-receptor affinity. The sole compound **21**, bearing a fluorine atom, conserved a similar receptor affinity (*K*_i_ = 65 nM) when compared to parental compound **20**. While derivatives **22** and 23 showed *K*_i_ values higher than 1 μM.

Concerning the virtual structural stiffening of cinnamyl chain, by introducing three different heterocyclic systems on DTD templates, only the indolic ring seems to be positive for this class of compounds, resulting in derivative **53** endowed with the high μ-receptor affinity, being the best among all compounds herein reported. By molecular docking mechanism assessment, arises a stronger interaction with the target binding site due to the nitrogen atom of the indole moiety that acts as hydrogen bond donor for Asp147.

In conclusion, this work evidenced as the flexibility of the cinnamyl chain is a prerequisite for the μ-receptor affinity of these derivatives, whereas its constriction in benzo-condensed or hetero-bicyclic system seems responsible for its selectivity for the same receptors. This work also showed a series of new derivatives endowed with an increased selectivity towards μOR associated with a lower μOR affinity. Further investigations will be carried out to synthesize new benzocondensed derivatives with a higher μOR affinity.

## Figures and Tables

**Table 1:**
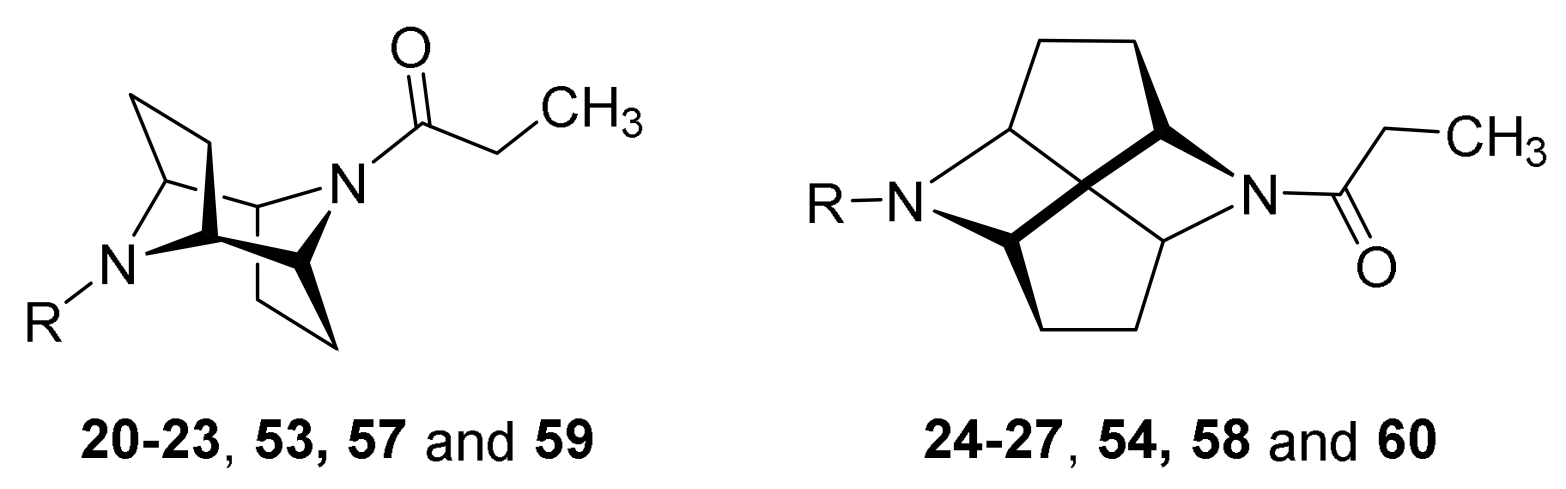
Binding affinity of DTDs **20**–**27**, **53**–**54** and **57**–**60** for opioid receptors.

Compound ^a^	R	R_1_		*K*_i_ (nM) ^b^	
			μ	δ	κ
**20**	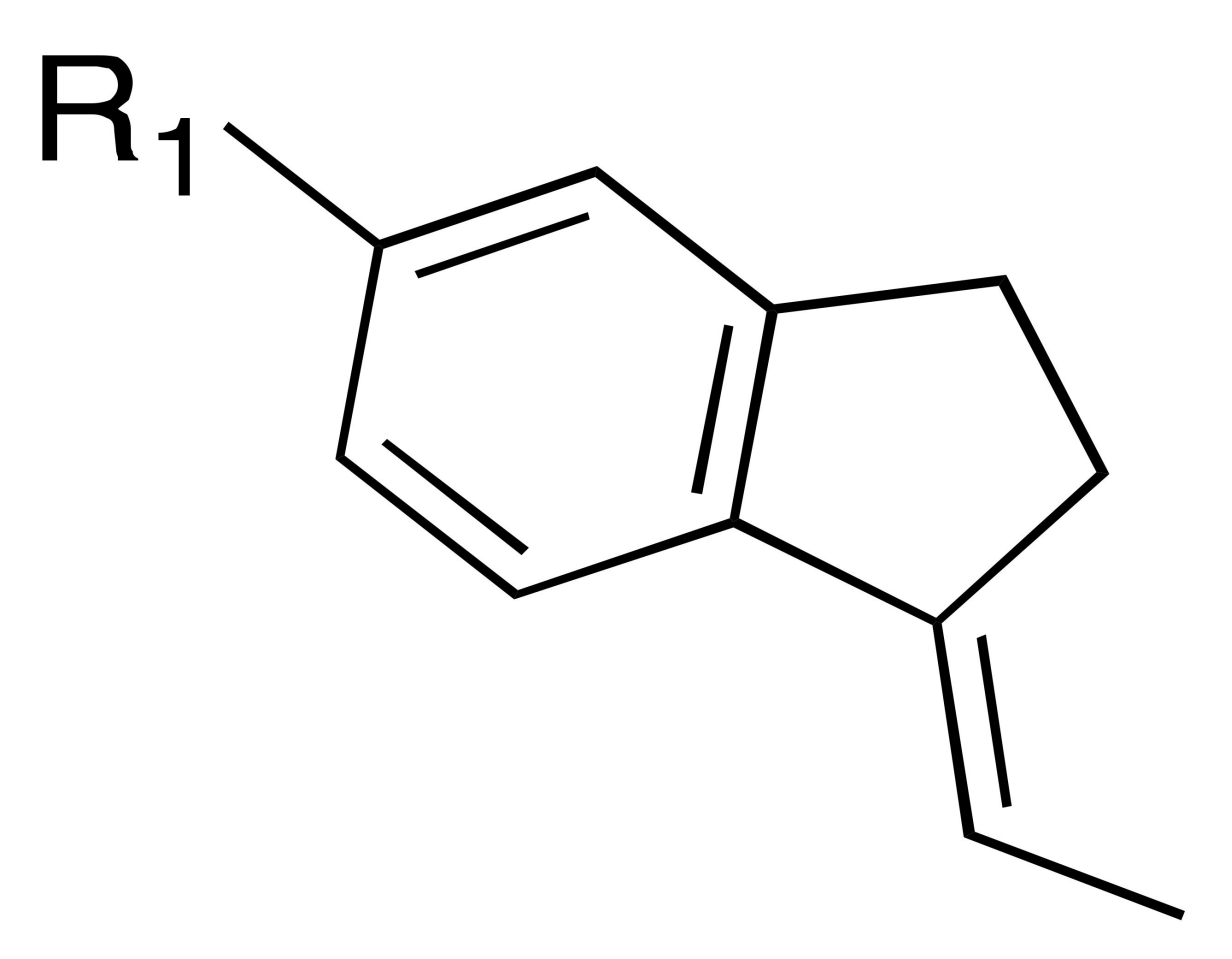	H	50 ± 4	>1000	>1000
**21**		F	65 ± 7	>1000	>1000
**22**		Cl	>1000	>1000	>1000
**23**		Br	>1000	>1000	>1000
**24**		H	100 ± 11	>1000	>1000
**25**		F	N.D.	N.D.	N.D.
**26**		Cl	75 ± 7	>1000	>1000
**27**		Br	200 ± 21	>1000	>1000
**53**	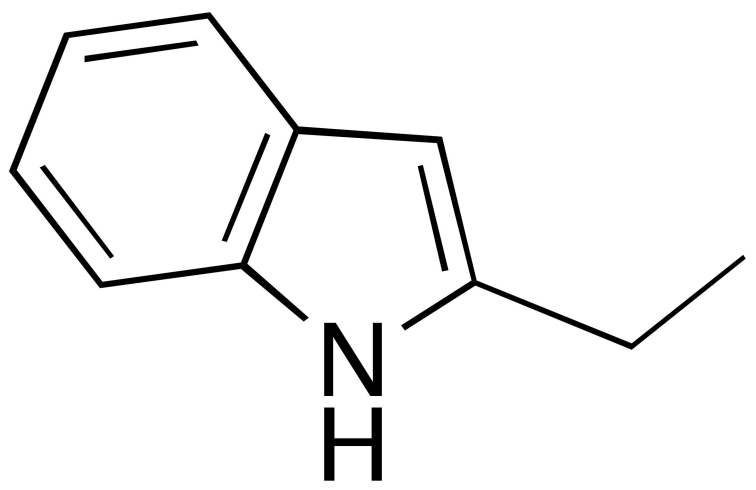	-	22 ± 3	>1000	>1000
**54**		-	500 ± 45	>1000	>1000
**57**	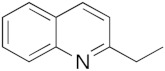	-	N.D. ^c^	N.D.	N.D.
**58**		-	490 ± 46	>1000	>1000
**59**	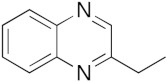	-	>1000	>1000	>1000
**60**		-	N.D.	N.D.	N.D.
**Morphine**			3.35 ± 0.30	100.2 ± 5.1	280.8 ± 9.2

^a^ The receptor binding affinities of all compounds were carried on their hydrochlorides. ^b^ The *K*_i_ was calculated from the IC_50_ values determined from the binding curves, using the Cheng-Prusoff equation. Values are the mean ± SEM of at least three independent experiments run in triplicate. ^c^ N.D. = not determined.

## Data Availability

All the data are reported in this paper.

## Sample Availability

Samples of the compounds are available from the authors.
